# A Comprehensive Study of Biohopanoid Production in *Alphaproteobacteria*: Biosynthetic, Chemotaxonomical, and Geobiological Implications

**DOI:** 10.1111/gbi.70038

**Published:** 2025-11-04

**Authors:** Jaap S. Sinninghe Damsté, Michel Koenen, Vera Thiel, Nora Richter, Ellen C. Hopmans, Nicole J. Bale

**Affiliations:** ^1^ Department of Marine Microbiology and Biogeochemistry NIOZ Royal Institute for Sea Research Texel the Netherlands; ^2^ Department of Earth Sciences, Faculty of Geosciences Utrecht University Utrecht the Netherlands; ^3^ Department of Microorganisms Leibniz‐Institute DSMZ–Deutsche Sammlung von Mikroorganismen und Zellkulturen Braunschweig Germany

**Keywords:** 2‐methyl hopane index, bacteriohopanepolyol derivatives, biosynthesis, biosynthetic gene clusters, hopanoids, methylated hopanoids, tetrahymanol

## Abstract

Bacteriohopanepolyol derivatives (BHPDs) and dia‐ and catagenetic products formed from these bacterial membrane modifiers are extensively used as biomarkers in molecular ecological and geoscience studies. Some BHPDs can be assigned to specific phylogenetic bacterial groups. With the vastly increasing availability of complete bacterial genomes, hopanoid production can be readily predicted by the presence of specific genes encoding biosynthetic enzymes involved in their production, but the time‐consuming physiological confirmation remains a critical element in the interpretation of such data in a biosynthetic and paleontological context. *Alphaproteobacteria* (APB) have often been proposed as important BHPD producers in a wide variety of environments and produce indicative BHPDs containing an additional methyl group in the A‐ring, complicating the assignment of 2‐methyl hopanes to N_2_‐fixing cyanobacteria in paleontological studies. Here we provide the first comprehensive study of the relationship between genotype and phenotype with respect to the production of C_30_ hopanoids and BHPDs by APB. Genome analysis of > 6000 reference genomes of APB revealed that ca. 23% possess the genetic capacity to produce BHPDs, which is substantially higher than for all bacteria. However, BHPD biosynthesis genes were unevenly distributed between taxonomic and phylogenetic groups and not consistently found in mono‐phylogenetic clusters. To study the relationship of genotype and phenotype with respect to the production of BHPDs, we cultivated 52 strains (50 terrestrial and 2 marine species) of the three major orders of the APB: *Hyphomicrobiales*, *Rhodospirillales*, and *Sphingomonadales*. These include species of 29 genera that have not previously been examined for BHPDs. Intact BHPDs were analyzed by UHPLC‐MS^n^, resulting in the identification of overall 63 different structures and a wide variety in BHPD distributions. These results were in line with those obtained from Rohmer degradation on intact cells, which were specifically used to accurately assess the degree of methylation at C‐2 and C‐3 of ring A of the BHPDs. This revealed a 1–2 orders of magnitude lower degree of methylation at C‐2 of BHPDs than for tetrahymanol (which was detected in three species all belonging to the *Nitrobacteraceae*) and C_30_ hopanoids, which has important implications for the interpretation of the molecular fossil record. Our results also showed that the presence of BHPD biosynthetic genes, often organized in a biosynthetic gene cluster, in all cases results in actual production of BHPDs. Thus, the presence of BHPD genes is a good predictor for the actual production of BHPDs. However, the presence of genes encoding proteins that result in methylation at C‐2 and C‐3 of BHPDs does not always lead to the production of methylated BHPDs, complicating the interpretation of the presence of the *hpnP* and *hpnR* genes in their genomes. Rohmer degradation‐derived BHPD concentrations in APB species that do produce hopanoids can vary by two orders of magnitude and are not directly related to a specific phylogenetic group, indicating that the origin of sedimentary BHPDs may be biased towards specific species that produce relatively high amounts of BHPDs. These findings constrain the use of BHPDs as biomarkers for specific groups of bacteria in environmental and palaeontological studies.

## 1 Introduction

**FIGURE 1:**
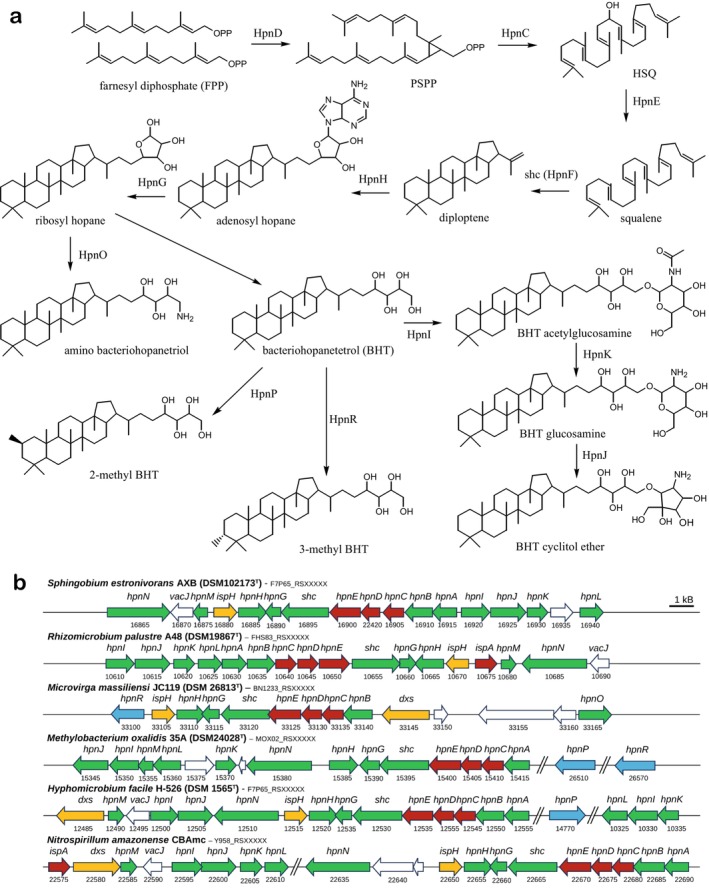
Biosynthesis of BHPDs by APB. (a) The building block of BHPDs, isoprenyl orthopyrophosphate (OPP), is synthesized by the proteins encoded by the genes of the MEP pathway (*dxs*, *dxr*, *ispD*, *ispE*, *ispF*, *ispG*, *ispH*; Zhao et al. 2013; Table S2, not shown in the scheme). Farnesyl OPP is produced by a reaction catalyzed by farnesyl diphosphate synthase encoded by *ispA* (not shown) (Thulasiram and Poulter 2006). The next step in the production of BHPDs is the synthesis of squalene by the coupling of two molecules of farnesyl diphosphate. This proceeds via a three‐step mechanism catalyzed by three different enzymes encoded by *hpnD*, *hpnC*, and *hpnE* (Pan et al. 2015), the ancestral pathway in bacteria (Santana‐Molina et al. 2020). The cyclization of squalene to form the C_30_ hopanoid building block diploptene is catalyzed by squalene‐hopene cyclase encoded by *shc* (Reipen et al. 1995; Kannenberg et al. 1996). The radical SAM adenosylhopane synthase, HpnH, encoded by *hpnH*, catalyzes the addition of the 5′‐deoxyadenosyl radical to diploptene and represents the first step in the biosynthesis of C_35_ BHPDs (Bradley et al. 2010; Sato et al. 2020, 2021). Subsequently, the adenine moiety is removed by the putative phosphorylase HpnG encoded by *hpnG* (Bradley et al. 2010; Liu et al. 2014), generating ribosylhopane. This intermediate can be converted into aminobacteriohopanetriol by the aminotransferase family protein HpnO encoded by *hpnO* (Welander et al. 2012; Liu et al. 2014). Alternatively, ribosylhopane is converted into BHT for which the responsible enzyme has not yet been established (Bodlenner et al. 2015). The formation of the BHT cyclitol ether (CE) from BHT proceeds in three steps (Schmerk et al. 2015). Firstly, BHT is converted into BHT N‐acetylglucosamine by the glycosyltransferase HpnI encoded by *hpnI*. Subsequently, the acetyl group is removed by the deacetylase HpnK encode by *hpnK*. Lastly, HpnJ, a member of the radical SAM superfamily, encoded by *hpnJ* results in the formation of the BHT CE. Ring‐A methylation of BHPDs occurs by the HpnP and HpnR proteins encoded by *hpnP* (Welander et al. 2010) and *hpnR* (Welander and Summons 2012). They also methylate diploptene and diplopterol (and tetrahymanol). The *hpnN* gene encodes an integral membrane protein, HpnN, which is a member of family of transporters and responsible for shuttling hopanoids to the outer membrane (Doughty et al. 2011; Kumar et al. 2017). HpnM is possibly involved in transport of hopanoids, especially at low salinity (Schmerk et al. 2015; Rubiano‐Labrador et al. 2015). The function of the *hpnA*, *hpnB*, and *hpnL* genes has not yet been established (Table S1). (b) The composition of the *hpn* BGCs or “islands” with the identified genes related to BHPD synthesis indicated. Examples for APB species of each of the four different orders of APB studied are given. If the gene cluster is dissected in pieces this is indicated by a double slash. The five‐number code below the genes refers, in combination with the code following the species name, to the locus tag in the annotated genome from the NCBI database. These numbers typically increase by 5 for every next gene. Color codes of genes are: dark yellow, genes involved in the mevalonate pathway of isoprenoid biosynthesis; red, genes involved in the biosynthesis of squalene; green, (putative) genes involved in BHPD biosynthesis; light blue, genes involved in the methylation of BHPDs; white, genes probably not involved in BHPD synthesis.

Hopanoids are one of the few natural products that were first identified in the geosphere before it was demonstrated that many bacteria produce them as so‐called “sterol‐surrogates” (Ourisson et al. 1979, 1987). Ever since, a wealth of scientific research has been performed on this interesting class of membrane lipids from both a biological and a paleontological perspective (see Belin et al. 2018 and Kusch and Rush 2022 for recent reviews). Giant steps were made in the last decades of the previous century in the structural elucidation of complex bacteriohopanepolyol derivatives (BHPDs) occurring in various genera of bacteria (see Rohmer 1993 for a review). Subsequently, the biosynthetic pathways of BHPDs together with the proteins catalyzing key reactions (Figure 1a) were elucidated step by step (e.g., Reipen et al. 1995; Bradley et al. 2010; Welander et al. 2012; Pan et al. 2015). This data now allow, in principle, the prediction as to whether a bacterium can produce BHPDs and, to some extent, in which forms based on its genomic composition. However, for many of the BHPDs, we still lack knowledge concerning specific steps of their biosynthesis (see Figure 1a).

From a molecular paleontological perspective, the omnipresent hopanoids have been intriguing components as well. They occur from diagenetically unaltered BHPDs in recent sediments to saturated hopanes in petroleum (see Kusch and Rush 2022 for a review), and the dia‐ and catagenetic pathways resulting in these transitions have been studied in detail (e.g., Sinninghe Damsté et al. 1995; Synnott et al. 2021). Ring‐A methylated hopanoids have played an important role in evolutionary and paleo‐reconstruction studies. For example, Summons et al. (1999) used the sedimentary record of 2‐methylhopanoids to constrain the advent of oxygenic photosynthesis by cyanobacteria. Some, but not all, present‐day cyanobacteria produce abundant 2‐methyl BHPDs (Summons et al. 1999; Talbot et al. 2008; Sáenz et al. 2012) and were thought to be important sources for sedimentary 2‐methylhopanoids. Kuypers et al. (2004) interpreted the high abundance of extended 2‐methyl hopanoids in black shales in conjunction with the sedimentary ^15^N content to indicate that cyanobacterial N_2_‐fixation was the main source of nutrient N in the stratified oceans of the Cretaceous oceanic anoxic events (OAEs). These interpretations were weakened by the reported presence of 2‐methyl BHPDs in an anoxygenic photoautotroph belonging to the *Alphaproteobacteria* (APB), *Rhodospeudomonas palustris*, which can produce 2‐methyl BHPDs under both aerobic and anaerobic conditions (Rashby et al. 2007). Subsequently, Welander et al. (2010) showed, by identification of the gene encoding the SAM radical protein responsible for the methylation at C‐2 (*hpnP*), that the potential to produce 2‐methyl BHPDs also occurs in other APB as well as in *Acidobacteria*. Recently, however, it was argued, based on the topology of an extended *hpnP* tree, that the *hpnP* gene appeared earlier in cyanobacteria than in APB and, thus, that 2‐methyl hopanes can be confidently used as markers for cyanobacteria in sediments older than 750 Ma (Hoshino et al. 2023). Elling et al. (2020) proposed that the abundance of 2‐methyl hopanoids during Cretaceous OAEs was caused by the abundance of members of the genus *Nitrobacter* spp. (nitrite‐oxidizing APB), which produce high amounts of 2‐methyl hopanoids when supplied with cobalamin (vitamin B_12_). Instead of methylation at C‐2, BHPDs can also be methylated at C‐3. 3‐Methyl BHPDs and their diagenetic products have been postulated as markers for aerobic methanotrophic bacteria (see Kusch and Rush 2022 for a review). Again, some APB are one of the known producers of 3‐methyl hopanoids (Rohmer 1993).

From these earlier studies, it has become clear that APB are considered important (methyl)hopanoid producers in the environment. Indeed, APB occur in a wide range of environments including soil as well as the water column and sediments of oceans and lakes. APB often represent a group in microbial communities that is abundant and metabolically active (Brinkhoff et al. 2008; Bates et al. 2011; Schiaffino et al. 2016). Gram‐negative APB are metabolically versatile. For instance, most members are chemo‐organoheterotrophs, but others perform anoxygenic photosynthesis (Brinkmann et al. 2018), including the families *Rhodobacteraceae* and *Rhodospirillaceae*, the so‐called purple non‐sulfur bacteria (Imhoff et al. 1998). Other APB are capable of aerobic methane oxidation (the so‐called Type II methanotrophs; Hakobyan and Liesack 2020) or nitrogen fixation (Rahimlou et al. 2021). The latter APB occur often in association with the roots of legumes in soil (e.g., *Rhizobium* spp.). The taxonomy of APB has recently been revised by analysis of > 1000 genomes of type strains of APB (Hördt et al. 2020). The major orders are the *Hyphomicrobiales*, *Rhodospirillales*, and *Sphingomonadales*, which contain the most described species.

APB species have often been used in the structural identification of BHPDs (see Rohmer 1993 for a review) and to elucidate the proteins catalyzing reactions in the biosynthesis of these BHPDs and the genes encoding them (Figure 1a; see legend for references). However, these studies have often been focused on easy‐to‐cultivate APB of genera, for example, members of the genera *Rhodopseudomonas*, *Methylobacter*, *Acetobacter*, and *Zymomonas*. Here we comprehensively investigate the genomic potential of hopanoid and BHPD production by APB in > 6000 strains and test hopanoid and BHPD production in 54 cultivated species using a state‐of‐the‐art UHPLC‐MS^n^ technique (Hopmans et al. 2021), which allowed for the identification of a wide variety of BHPDs. We discuss our findings with respect to both their biochemical and geobiological implications.

## 2 Materials and Methods

### 2.1 Cultivation

A selection of 52 strains were cultured for biomass production and subsequent BHPD analyses (Table 1). Cultures were grown at DSMZ in 0.5 or 1 L Erlenmeyer flasks or on agar plates, using the respective media and cultivation conditions given in the DSMZ online catalog (see https://www.dsmz.de/collection/catalogue/microorganisms/catalogue). Biomass was harvested in late exponential or early stationary phase. The cells were collected by centrifugation in either a Sorvall RC 6plus centrifuge (rotor F109‐6x500y or F21‐8x50) at 9000 rpm for 20 min, a Heraeus Multifuge 1S‐R (rotor type 2001), or in a Beckman coulter centrifuge Avanti J‐30I (rotor JA‐14; Galway, IRE) at 10000 rpm for 20 min. After the liquid was removed, the harvested cells were lyophilized in a freeze‐drying machine Christ Alpha 1–4 LDplus (Osterode am Harz, GER) at −45°C and 0.04 mbar.

**TABLE 1:** Relevant information on the 54 species of APB studied for production of BHPDs in this study.

Order	Family	Species–strain number	DSM culture	Origin	Literature^a^	Genome assembly^b^
*Rhodospirillales*	*Acetobacteraceae*	*Muricoccus pecuniae* N75	25622^T^	Surface of a copper European coin (Portugal)	1	GCF_014199205.1
		*Rhodopila globiformis* 7950	161^T^	Sulfur spring (Yellowstone, USA)	2	GCF_002937115.1
		*Acidocella aminolytica* 101	11237^T^	Acidic mine drainage (Japan)	3	GCF_900129125.1
		*Gluconacetobacter diazotrophicus* PA 5	5601^T^	Sugarcane roots (Brazil)	4	GCF_000021325.1
		*Komagataeibacter xylinus* R‐2277	n.a.	Unknown	5	GCF_000964505.1^c^
		*Komagataeibacter europaeus* JK2	13110	Cider vinegar (Slovenia)	6	GCF_002173515.1^d^
		*Kozakia baliensis* YO‐3	14400^T^	Palm brown sugar (Indonesia)	7	GCF_001787335.1
		*Asaia siamensis* S60‐1	15972^T^	Flower of *Calotropis gigantea* (Thailand)	8	GCF_014635085.1
		*Gluconobacter oxydans* CN 1221	2003	Unknown	9	GCF_000507285.1
		*Acetobacter pasteurianus* 190	3509^T^	Beer	10	GCF_003850805.1
	*Rhodovibrionaceae*	*Limimonas halophila* IA16	25584^T^	Mud from a hypersaline lake (Iran)	11	GCF_900100655.1
		*Pelagibius litoralis* CL‐UU02	21314^T^	Seawater (Korea)	12	GCF_011683915.1
		*Tistlia consotensis* USBA 355	21585^T^	Saline spring (Colombia)	13	GCF_900188055.1
	*Rhodospirillaceae*	*Rhodospirillum rubrum* S 1	467^T^	Unknown	14	GCF_019134555.1
		*Magnetospirillum fulvum* 1360	113^T^	Sewage pond	15	GCF_900108475.1^e^
	*Reyranellaceae* ^f^	*Enhydrobacter aerosaccus* G	8914^T^	Wintergreen Lake (USA)	16	GCF_900167455.1
	*Rhodospirillaceae*	*Hypericibacter terrae* R5913	109816^T^	Soil of *Hypericum perforatum* (Germany)	17	GCF_008728855.1
	*Azospirillaceae*	*Skermanella aerolata* 5416 T‐32	18479^T^	Air (South Korea)	18	GCF_000936425.1
		*Nitrospirillum amazonense* Y‐1	2787^T^	*Digitaria decumbens* roots (Brazil)	19	GCF_029594735.1
		*Azospirillum brasilense* Sp 7	1690^T^	*Digitaria decumbens* roots (Brazil)	20	GCF_008274945.1
*Sphingomonadales*	*Erythrobacteraceae*	*Novosphingobium nitrogenifigens* Y88	19370^T^	Pulp and paper waste water (New Zealand)	21	GCF_000192575.1
		*Novosphingobium acidiphilum* FSW 06‐204d	19966	Bog lake Grosse (Germany)	22	GCF_000429005.1
		*Novosphingobium rosa* R13*5*	7285^T^	Rose	23	GCF_001598555.1
	*Sphingomonadaceae*	*Sphingomonas mali* Y‐347	10565^T^	Roots of apple tree (Japan)	24	GCF_001598415.1
		*Stakelama sediminis* CJ70	27203^T^	Tidal flat sediment (South Korea)	25	GCF_014199335.1
		*Sphingomonas alpina* S8‐3	22537^T^	Alpine soil (Austria)	26	GCF_014490665.1
		*Sphingomonas changbaiensis* V2M44	25652^T^	Forest soil (China)	27	GCF_000974765.1
		*Sphingomonas formosensis* CC‐Nfb‐2	24164^T^	Oil contaminated soil (Taiwan)	28	GCF_009755815.1
		*Sphingomonas haloaromaticamans* A175	13477^T^	Water and soil (Netherlands)	29	GCF_001853345.1
		*Sphingobium estronivorans* AXB	102173^T^	Sewage plant (China)	30	GCF_008692605.1
*Micropepsales*	*Micropepsaceae*	*Rhizomicrobium palustre* A48	19867^T^	Roots of rice plants *Oryza sativa* (Japan)	31	GCF_011761565.1
*Hyphomicrobiales*	*Rhizobiaceae*	*Rhizobium tropici* HAMBI 1163	11418^T^	Root nodule of *Phaseolus vulgaris* (South America)	32	GCF_000330885.1
		*Ensifer sojae* CCBAU 05684	26426^T^	Effective nodules of *Glycine max* (China)	33	GCF_002288525.1
	*Stappiaceae*	*Roseibium album* 50 M6	18320^T^	Oyster meat (Spain)	34	GCF_001404515.1
		*Roseibium marinum* mano 18	17023^T^	Seawater (Korea)	35	GCF_002906165.1
	*Parvibaculaceae*	*Rhodoligotrophos appendicifer* 120–1	23582^T^	Freshwater lake (Antarctica)	36	GCF_007474605.1
	*Hyphomicrobiaceae*	*Rhodomicrobium vannielii* ATH 3.1.1	162^T^	Unknown	37	GCF_000166055.1
		*Hyphomicrobium facile* H‐526	1565^T^	Soil (USA)	38	GCF_900116175.1
	*Chelatococcaceae*	*Chelatococcus reniformis* JCM 30308	105737^T^	Ice core of glacier (China)	39	GCF_014640075.1
	*Methylobacteriaceae*	*Enterovirga rhinocerotis* YIM 100770	25903^T^	*Rhinoceros unicornis* faeces (China)	40	GCF_004363955.1
		*Microvirga massiliensis* JC119	26813^T^	Human feces (Senegal)	41	GCF_001006805.1
		*Methylobacterium soli* YIM 48816	21955^T^	Forest soil (China)	42	GCF_008806385.1
		*Methylobacterium oxalidis* 35a	24028^T^	Leaves of *Oxalis corniculata* (Japan)	43	GCF_022179505.1
		*Methylorubrum rhodesianum* D2_2	103741	Cleanroom facility (Italy)	44	GCF_014199985.1^g^
		*Methylobacterium aquaticum* GR16	16371^T^	Drinking water (Spain)	45	GCF_001043915.1
	*Roseiarcaceae*	*Roseiarcus fermentans* Pf56	24875^T^	Acidic peat soil of a *Spagnum* peat bog (Russia)	46	GCF_003315135.1
	*Beijerinckiaceae*	*Methyloferula stellata* AR4	22108^T^	Acidic peat soil of a fen (Russia)	47	GCF_000385335.1
		*Methylocella palustris* K	n.a.	Acidic *Sphagnum* peat bog (Russia)	48	GCF_038024855.1^h^
		*Beijerinckia indica* B.102.C	591	Sugar cane soil (Brazil)	49	GCF_000019845.1^i^
	*Nitrobacteraceae*	*Rhodoplanes elegans* AS130	11907^T^	Activated sludge	50	GCF_016653355.1
		*Variibacter gotjawalensis* GJW‐30	29671^T^	Forest soil (South Korea)	51	GCF_004216635.1
		*Afipia broomeae* B91‐007286	7327^T^	Sputum (New Zealand)	52	GCF_000314675.2
		*Rhodopseudomonas parapalustris* Dr.1	130	Ditch	53	GCF_000013365.1^j^
		*Bradyrhizobium elkanii* USDA61	11554^T^	Soybean *Glycine max* (USA)	54	GCF_012871055.1

^a^ Literature quoted: Note that reference is given to papers describing the first isolation and not to papers where their taxonomy was reassigned. (1) Lopes et al. (2011); (2) Pfennig (1974); (3) Kishimoto et al. (1993); (4) Gillis et al. (1989); (5) Yamada et al. (2012); (6) Sievers et al. (1992); (7) Lisdiyanti et al. (2002); (8) Katsura et al. (2001); (9) Leisinger (1965); (10) Hansen (1879); (11) Amoozegar et al. (2013); (12) Choi et al. (2009); (13) Díaz‐Cárdenas et al. (2010); (14) and (15) Pfennig and Trüper (1971); (16) Staley et al. (1987); (17) Noviana et al. (2020); (18) Weon et al. (2007); (19) Magalhaes et al. (1983); (20) Tarrand et al. (1978); (21) Addison et al. (2007); (22) Glaeser et al. (2009); (23) and (24) Takeuchi et al. (1995); (25) Thawng et al. (2013); (26) Margesin et al. (2012); (27) Zhang et al. (2010); (28) Lin et al. (2012); (29) Wittich et al. (2007); (30) Qin et al. (2020); (31) Ueki et al. (2010); (32) Martínez‐Romero et al. (1991); (33) Li et al. (2011); (34) Pujalte et al. (2005); (35) Kim et al. (2006); (36) Fukuda et al. (2012); (37) Duchow and Douglas (1949); (38) Hirsch and Conti (1964); (39) Gu et al. (2016); (40) Chen et al. (2017); (41) Caputo et al. (2016); (42) Cao et al. (2011); (43) Tani et al. (2012); (44) Green and Ardley (2018); (45) Gallego et al. (2005); (46) Kulichevskaya et al. (2014); (47) Vorobev et al. (2011); (48) Dedysh et al. (2000); (49) Starkey and De (1939); (50) Hiraishi and Ueda (1994); (51) Kim et al. (2014); (52) Brenner et al. (1991); (53) Ramana et al. (2012); (54) Kuykendall et al. (1992).

^b^ NCBI RefSeq assembly.

^c^ No genome available for strain R‐2277. The genome of *K. xylinus* NBRC 15237 was used instead.

^d^ No genome available for strain JK2. The genome of *K. europeus* SRCM101446 (reference genome) was used instead. The 16S rRNA gene of strain Y15289 is 99.93% identical to that of strain SRCM101446.

^e^ No genome available for strain 1360. The genome of *M. fulvum* DSM13234 was used instead.

^f^ Based on the sequence similarity of the 16S rRNA gene and the gyrB protein, *E. aerosaccus* is most closely affiliated with *Reyranella* sp.

^g^ No genome available for strain D2_2. The genome of *M. rhodesianum* DSM 5687^T^ (reference genome) was used instead.

^h^ No genome available for strain K. The genome of *M. silvestris* BL2 (DSM 15510^T^) was used instead. The 16S rRNA gene of strain K is 96.99% identical to that of strain BL2 and 97.85% identical to that of *M. tundrae* T4T (DSM 15673^T^). The genomes of *M. silvestris and M. tundrae* have an identical distribution of *hpn* genes, suggesting that both would be representative of that of *M. palustris* .

^i^ No genome available for strain B.102.C. The genome of *B. indica* DSM 1715^T^ (reference genome) was used instead.

^j^ No genome available for strain Dr.1. The genome of *R. palustris* HaA2 was used instead. The 16S rRNA gene of strain Dr.1 is 99.35% identical to that of strain HaA2.

### 2.2 Analysis of Intact BHPDs

The lyophilized bacterial biomass was extracted at NIOZ using a modified Bligh‐Dyer extraction (Bale et al. 2021). Analysis of intact composite BHPD was subsequently carried out by direct analysis following the method of Hopmans et al. (2021), which allows the identification and semi‐quantitation of non‐derivatized BHPD by ultra‐high performance liquid chromatography‐electrospray ionization‐high resolution multistage mass spectrometry (UHPLC‐HRMS^n^). Fragmentation mass spectra provided information on the BHPD core, functionalized side chain, as well as the conjugated moiety of composite BHPDs.

### 2.3 Rohmer Degradation of Intact Cells

For the indirect detection of BHPDs and their quantification, lyophilized cells were directly treated with periodic acid/sodium borohydride to convert BHPDs into GC‐amenable hopanoid alcohols (hopanols) following the approach of Sinninghe Damsté et al. (2017). For experimental handling, we used procedure 2 originally described by Rohmer et al. (1984) with some modifications. To ground lyophilized cells (8–30 mg), 150 μL of a solution of 0.205 mg mL^−1^ of the internal standard (*n*‐tricosan‐1‐ol) was added for quantification purposes and subsequently stirred with a solution of periodic acid (30 mg) in 1 mL tetrahydrofuran/water (8:1, v/v) at room temperature for 1 h. The lipids were extracted three times from the reaction mixture with dichloromethane (2 mL) after the addition of 1 mL deionized water. Subsequently, the solution was dried over anhydrous Na_2_SO_4_, evaporated to dryness, and dissolved in 1 mL methanol. This mixture was subsequently treated with 20 mg of NaBH_4_ by stirring at room temperature for 1 h. After the addition of 1 mL of 200 mM KH_2_PO_4_, the hopanols formed by cleavage of the side‐chain of the BHPDs (i.e., hopan‐30‐ol, homohopan‐31‐ol, and bishomohopan‐32‐ol, and their unsaturated counterparts), and other extractable lipids unaffected by the Rohmer degradation reaction were extracted with dichloromethane. The extract was methylated with diazomethane, silylated with N,O‐bis(trimethylsilyl)‐fluoroacetamide in pyridine at 60°C for 20 min, and analyzed by GC and GC–MS. GC and GC–MS analysis were performed as described previously (Bale et al. 2019). The distribution and quantification of hopanols and other lipids were obtained by integration of the appropriate peaks and that of the internal standard.

### 2.4 GC–MS/MS Analysis of the Degree of Methylation of C_30_ Hopanoids, Tetrahymanol, and Rohmer Degradation Products

To quantify the presence of 2‐methyl (typically co‐eluting with the non‐methylated counterpart) and 3‐methyl derivatives of C_30_ hopanoids, tetrahymanol, and Rohmer degradation products, GC–MS/MS analysis was performed on an Agilent 7890B GC system interfaced to a 7000 C/Triple Quadrupole MS, operated in multiple reaction monitoring (MRM) mode. The oven program, column, carrier gas, and MS settings were the same as for GC–MS, apart from the interface temperature, which was 330°C and a runtime of 60.5 min (kept for solely 10 min at 320°C). Transitions from the molecular ion, in the case of hopenes, or the *m/z* M^+^.−90 ion formed by the loss of Si(CH_3_)_3_OH, in the case of silylated alcohols, to *m/z* 191 and 205 were measured in four time windows (Table S1) to accurately determine the degree of methylation of the hopenes and (extended) triterpenoid alcohols.

### 2.5 Bioinformatics Analyses

Biosynthetic genes involved in the methylerythritol phosphate (MEP) pathway of isoprenoid biosynthesis, squalene synthesis and cyclisation, hopanoid methylation, and the BHPD pathway were identified in APB genomes (see Table 1 for genome assembly numbers) with manual Position‐Specific Iterated BLAST (PSI‐BLAST) searches at the protein level with the NCBI Protein Reference Sequences database (Haft et al. 2023) using the NCBI website. A 1–3 iteration steps process was conducted using the annotated enzymes of *Rhodopseudomonas palustris* TIE‐2 or, when unavailable, other species (see Table S2) as query sequences in October 2023. BHPD biosynthesis genes have been previously annotated in various APB, that is, *Zymomonas mobilis* ZM4 (Reipen et al. 1995; Perzl et al. 1998), *R. palustris* TIE‐2 (Welander et al. 2010, 2012; Neubauer et al. 2015), *Bradyrhizobium diazoefficiens* USDA110 (Kannenberg et al. 1996; Perzl et al. 1998), *Methylobacterium extorquens* Bath (Bradley et al. 2010), in a betaproteobacterium, *Burkholderia cenocopacia* K56‐2 (Schmerk et al. 2015), and in a gammaproteobacterium, *Methylococcus capsulatus* (Welander and Summons 2012).

To generate a 16S rRNA gene tree, 55 sequences available in the NCBI database (Table S3) were retrieved from a local NCBI's NT (Sayers et al. 2024) database using blastdbcmd. Sequences were aligned using MAFFT X‐INS‐i v7.407 using default settings (Katoh et al. 2002) and trimmed using BMGE V1.12 (settings: ‐h 0.55 ‐t DNA) (Criscuolo and Gribaldo 2010). The alignment was used to reconstruct a maximum likelihood phylogenetic tree using IQ‐TREE v2.1.1 (settings: ‐m MFP) (Nguyen et al. 2015). Subsequently, the resultant tree was visualized and edited using iTOL v6 (Letunic and Bork 2024).

## 3 Results

### 3.1 General Indication for the Capacity of Hopanoid and BHPD Production by APB

A substantial part of the biosynthetic steps to produce BHPDs and the genes and enzymes involved are known (Table S2). The genes are typically referred to as *hpnX*, with *hpn* being the mnemonic for “hopanoid,” followed by a capital letter (X) signifying the actual gene. We searched for the corresponding proteins (HpnX) of APBs present in the NCBI reference sequence (RefSeq) database (Haft et al. 2023), which were at the time of the search predominantly derived from ca. 6700 genomes of pure or enriched APB cultures. This set of reference genomes is likely biased by a large contribution of genomes of APBs of interest for agricultural, ecological, food, or medical sciences and, thus, cannot be considered representative of all natural biomes on Earth. Genomes from marine APB species are especially underrepresented. In our search, we focused on the presence of squalene‐hopene cyclase (Shc; also referred to as HpnF), catalyzing the C_30_ hopanoid synthesis by cyclisation of squalene, HpnH (indicative of bacteriohopanetetrol [BHT] biosynthesis), and HpnP and HpnR (methylation at C‐2 and C‐3, respectively, of C_30_ hopanoids and BHPDs) (Figure 1a). In most cases, the HpnX proteins of *Rhodopseudomonas palustris* TIE‐1 were used as query sequences in PSI‐BLAST searches since their proteins catalyzing BHPD biosynthesis have been well described (Welander et al. 2010; Neubauer et al. 2015; Table S2). For taxonomic reference, we used the GyrB protein of *Rhodopseudomonas palustris* TIE‐1 (WP_012493855.1), catalyzing the synthesis of the B subunit of the DNA gyrase, a ubiquitous housekeeping protein for DNA repair in bacteria (Reece and Maxwell 1991). Because of its conservative nature, GyrB is commonly used as a phylogenetic marker to map the diversity of bacteria. PSI‐BLAST searches with GyrB of *Rhodopseudomonas palustris* TIE‐1, our model APB, resulted in > 6500 unique GyrB sequences distributed over the various orders of APBs: *Hyphomicrobiales* (2618 hits; 171 genera), *Rhodobacterales* (1337; 203 genera), *Rhodospirillales* (673; 106 genera), *Sphingomonadales* (1129; 51 genera), and other orders (753; 87 genera) (Table 2, Table S4). Some genera are overrepresented in this collection because of the high number of reference sequences available in the public online database due to their relevance for agricultural, ecological, food, or medical sciences: for example, *Acetobacter* (94 hits), *Bartonella* (116), *Bradyrhizobium* (366), *Brevundimonas* (188), *Methylobacterium* (139), *Mesorhizobium* (315), *Novosphingobium* (141), *Paracoccus* (146), *Rhizobium* (402), *Ruegeria* (106), *Sphinobium* (116), *Sphingomonas* (409), and *Wolbachia* (409).

**TABLE 2:** The potential of (ring‐A methylated) hopanoid biosynthesis by APB as revealed by the presence of key proteins (Shc, HpnH, HpnP, and HpnR) as encoded by > 6500 APB reference genomes in the NCBI database.

Order	Family	Genus^a^	GyrB	Sim (%)	Shc	Sim (%)	HpnH	Sim (%)	HpnP	Sim (%)	HpnR	Sim (%)	DSMZ
			#		#		#		#		#		Strains
*Caulobacterales*	*Caulobacteraceae*	*Caulobacter*	55	66.2 ± 1.0	3	63.2 ± 1.7	3	60.8 ± 0.3	—		—		19
		*Phenylobacterium*	61	66.4 ± 0.6	3	60.9 ± 1.0	3	63.1 ± 0.5	—		—		21
*Hyphomicrobiales* ^b^	*Amorphaceae*	*Acuticoccus*	7	70.5 ± 0.4	1	60.8	1	61.1	—		—		1
	*Aurantimonadaceae*	*Aureimonas*	31	69.8 ± 1.1	3	64.2 ± 0.8	3	61.4 ± 1.3	—		—		11
	*Beijerinckiaceae*	***Beijerinckia*** ^1^	4	70.9 ± 0.7	3	59.6 ± 0.9	3	57.1 ± 0.2	3	63.1 ± 0.6	—		14
		*Methylocapsa*	5	71.0 ± 0.2	4	61.5 ± 1.3	5	59.6 ± 1.1	5	62.5 ± 1.1	—		3
		***Methylocella*** ^2‐3^	4	72.1 ± 0.5	3	62.3 ± 0.7	3	60.3 ± 0.3	4	63.9 ± 0.4	—		2
		***Methyloferula***	1	72.0	1	63.6	1	60.7	1	63.9	—		1
		*Methylovirgula*	4	71.3 ± 1.2	3	63.0 ± 1.8	3	61.1 ± 0.9	2	62.7 ± 0.1	—		1
		*Rhodoblastus* ^4‐7^	10	73.3 ± 0.4	3	66.8 ± 2.1	11	62.8 ± 1.9	1	54.8			4
	*Nitrobacteraceae*	***Afipia***	14	86.1 ± 1.0	14	69.8 ± 3.5	13	87.1 ± 0.8	14	70.1 ± 1.8	—		10
		***Bradyrhizobium*** ^8‐10^	366	83.7 ± 1.9	410	77.1 ± 1.9	325	87.0 ± 1.1	412	73.6 ± 1.1	—		35
		*Nitrobacter* ^11^	8	87.6 ± 0.3	8	75.1 ± 0.8	7	87.4 ± 0.6	8	73.8 ± 0.8	—		4
		***Rhodopseudomonas*** ^5‐6,12‐14^	25	93.5 ± 4.0	26	89.3 ± 5.3	27	91.7 ± 4.9	29	90.5 ± 9.4	—		11
		*Tardiphaga*	11	87.9 ± 0.6	14	73.8 ± 0.7	11	85.2 ± 0.9	12	71.9 ± 1.6	—		3
		***Variibacter***	1	77.8	1	70.1	1	74.4	—		—		1
		*Pseudorhodoplanes*	1	79.0	1	68.6	1	82.3	—		—		—
	*Chelatococcaceaea*	***Chelatococcus***	9	75.6 ± 1.5	2	63.8 ± 0.7	2	59.9 ± 1.0	—		—		9
	*Hyphomicrobiaceae*	***Hyphomicrobium*** ^12^	25	68.4 ± 0.5	10	65.1 ± 0.8	9	61.8 ± 1.3	2	67.3 ± 0.7	—		12
		***Rhodomicrobium*** ^12,13,15^	7	70.0 ± 1.2	7	66.1 ± 1.9	5	59.9 ± 1.4	—		—		8
		***Rhodoplanes*** ^16^	7	79.8 ± 1.3	8	69.5 ± 0.6	8	76.3 ± 5.2	7	63.1 ± 0.1	—		5
	*Lichenibacteriaceaea*	*Lichenibacterium*	3	71.2 ± 0.6	6	60.0 ± 2.5	3	61.5 ± 0.7	—		—		—
	*Lichenihabitantaceae*	*Lichenifustis*	1	72.1	1	62.3	1	59.4	1	57.5	—		—
		*Lichenihabitans*	3	72.2 ± 0.1	5	61.7 ± 2.7	2	60.0 ± 0.9	3	58.2 ± 0.0	—		—
	*Methylobacteriaceae*	***Enterovirga***	3	68.1 ± 0.4	2	60.5 ± 0.7	3	59.2 ± 1.0	3	57.1 ± 0.3	—		1
		***Methylobacterium*** ^6,12,13,17‐20^	137	70.6 ± 0.9	95	63.0 ± 2.0	95	60.0 ± 0.7	132	59.3 ± 1.0	3	65.2 ± 1.4	105
		***Methylorubrum***	19	70.7 ± 0.3	26	61.3 ± 2.6	9	59.5 ± 0.4	17	60.4 ± 0.3	—		18
		***Microvirga***	45	73.8 ± 0.9	4	62.8 ± 1.6	2	60.5 ± 0.1	—		1	66.1	10
	*Methylocystaceae*	*Hansschlegelia*	4	75.3 ± 0.8	1	61.7	1	58.4	—		—		2
		*Methylocystis* ^12,21^	25	75.3 ± 0.8	23	64.0 ± 0.9	15	60.5 ± 0.7	4	55.3 ± 0.3	—		4
		*Methylosinus* ^2,12,21,22^	14	71.6 ± 0.6	13	65.3 ± 0.6	13	59.8 ± 0.8	—		—		10
	*Parvibaculaceaea*	*Kaustia*	1	74.1	1	56.2	1	62.8	—		—		—
		*Pyruvatibacter*	1	69.7	1	55.8	2	66.5 ± 0.2	—		—		—
		*Ca. Phaeomarinobacter*	1	69.8	1	77.3	1	66.1	—		—		—
		***Rhodoligotrophos***	2	71.3 ± 2.2	2	53.9 ± 0.5	2	64.6 ± 0.5	2	59.2 ± 1.0	—		1
	*Pleomorphomonadaceae*	*Faunimonas*	1	72.3	1	63.7	1	63.9	—		—		—
	*Reyranellaceae*	*Reyranella*	13	68.3 ± 0.8	5	63.7 ± 0.8	5	59.9 ± 1.4	—		1	59.4	3
	*Rhizobiaceae*	***Ensifer***	26	71.8 ± 0.4	2	76.1 ± 0.1	—		—		—		40
		*Sinorhizobium*	46	71.9 ± 0.5	4	75.9 ± 0.2	—		—		—		2
	*Roseiarcaceaea*	***Roseiarcus***	1	72.2	1	64.5	1	60.4	—		—		1
	*Salinarimonadaceae*	*Salinarimonas*	3	73.3 ± 1.5	1	55.2	1	64.5	—		—		2
	*Xanthobacteraceae*	*Labrys*	7	73.3 ± 0.4	3	65.4 ± 0.5	3	61.6 ± 0.7	—		—		8
		*Pseudolabrys*	3	77.0 ± 0.2	1	68.6	1	62.5	—		—		1
		*Starkeya*	4	73.8 ± 0.6	1	59.7	1	59.1	—		—		4
	*Incertae sedis*	***Enhydrobacter***	4	61.3 ± 8.5	2	63.8 ± 0.7	2	57.9 ± 0.4			—		1
		*Terrihabitans*	1	74.8	1	64.2	1	60.5	—		—		—
		*Vineibacter*	1	66.7	1	63.3	1	59.4	—		—		—
*Iodidimonadales*	*Iodidimonadaceae*	*Iodidimonas*	4	64.9 ± 0.4	4	56.3 ± 0.4	5	69.6 ± 0.8	—		—		—
*Micropepsales*	*Micropepsaceae*	***Rhizomicrobium***	2	70.3 ± 1.4	1	60.8	2	60.1 ± 1.1	—		—		4
*Minwuiales*	*Minwuiaceae*	*Minwuia*	2	66.2 ± 0.8	1	56.9	1	63.8	—		—		—
*Parvularculales*	*Parvularculaceae*	*Parvularcula*	9	66.9 ± 0.9	3	50.8 ± 0.5	2	67.6 ± 0.8	—		—		2
*Rhodobacterales* ^c^	*Rhodobacteraceae*	*Limibaculum*	2	67.3 ± 0.9	2	52.6 ± 1.2	1	68.7	—		—		1
		*Rhodovulum* ^23^	26	68.7 ± 2.2	1	70.6	1	76.6	1	61.3	—		9
		*Thermohalobaculum*	1	67.2	1	52.2	—		—		—		1
*Rhodospirillales* ^d^	*Acetobacteraceae*	***Acetobacter*** ^12,17,24–28^	94	60.2 ± 0.5	78	55.0 ± 0.5	63	62.0 ± 1.0	—		58	88.8 ± 4.7	8
		*Acidibrevibacterium*	2	60.4 ± 0.1	2	56.7 ± 0.1	1	66.5	—		2	60.9 ± 0.0	—
		*Acidiphilium*	7	59.7 ± 0.4	7	57.3 ± 0.7	7	60.1 ± 0.6	—		1	60.7	9
		*Acidisoma*	5	59.3 ± 0.4	4	58.1 ± 0.8	4	60.1 ± 0.3	—		—		4
		*Acidisphaera*	3	60.8 ± 0.8	3	60.2 ± 0.8	3	62.7 ± 1.2	—		—		1
		***Acidocella***	17	60.1 ± 0.7	15	57.8 ± 0.6	13	58.4 ± 1.5	—		—		4
		*Acidomonas*	2	60.7 ± 0.4	3	57.7 ± 0.9	2	60.6 ± 0.2	—		—		1
		*Ameyamaea*	1	60.7	1	57.0	1	60.5	—		—		—
		*Aristophania*	1	58.3	2	56.6 ± 0.0	1	58.8	—		—		—
		***Asaia***	12	61.3 ± 0.4	12	56.2 ± 0.2	8	60.9 ± 0.4	—		—		4
		*Belnapia*	6	60.5 ± 0.5	6	56.6 ± 1.0	6	59.2 ± 0.5	—		—		3
		*Bombella*	12	57.8 ± 0.3	14	55.0 ± 0.5	7	61.0 ± 0.6	—		—		3
		*Brytella*	1	59.7	1	56.4	1	60.1	—		—		—
		*Caldovatus*	2	62.6 ± 0.1	1	59.0	2	62.1 ± 0.1	—		—		—
		*Commensalibacter*	23	58.1 ± 0.4	20	55.1 ± 0.7	13	58.5 ± 0.5	—		—		—
		*Crenalkalicoccus*	1	60.5	1	55.9	1	61.2	—		—		—
		*Dankookia*	1	59.2	1	53.2	1	61.2	—		—		—
		*Endobacter*	1	60.5	1	56.4	1	60.8	—		—		—
		*Entomobacter*	1	57.8	1	57.7	1	59.8	—		1	73.3	1
		*Formicincola*	1	60.1	1	56.5	1	57.3	—		—		—
		***Gluconacetobacter*** ^12,13,24,29‐30^	15	59.8 ± 0.5	16	57.3 ± 1.7	12	61.6 ± 1.1	—		13	80.5 ± 1.4	5
		***Gluconobacter*** ^12^	52	58.2 ± 0.5	68	54.9 ± 1.0	19	60.2 ± 0.7	—		13	85.5 ± 0.2	15
		*Granulibacter*	5	59.4 ± 0.1	6	57.6 ± 0.1	3	62.3 ± 0.2	—		—		1
		***Komagataeibacter*** ^3,31^	33	60.1 ± 0.4	21	56.1 ± 0.4	21	60.5 ± 0.7	—		22	77.8 ± 0.3	22
		***Kozakia***	2	59.9 ± 0.2	3	57.2 ± 0.1	1	59.5	—		1	76.5	1
		*Lichenicoccus*	1	58.8	1	56.1	1	60.1	—		—		—
		*Lichenicola*	1	59.9	1	57.4	1	61.7	—		—		—
		*Limobrevibacterium*	1	61.1	1	58.7	1	62.0	—		—		—
		*Neoasaia*	1	60.0	1	56.7	1	59.0	—		—		—
		*Neokomagataea*	4	58.1 ± 0.1	4	56.3 ± 0.4	3	59.1 ± 0.2	—		—		—
		*Nguyenibacter*	1	59.9	1	58.6	1	62.7	—		—		—
		*Novacetimonas*	6	60.4 ± 0.3	10	55.6 ± 0.4	5	60.3 ± 0.7	—		6	77.5 ± 0.2	—
		*Oecophyllibacter*	3	59.4 ± 0.1	3	55.1 ± 0.1	1	61.1	—		—		1
		*Paracraurococcus*	2	59.9 ± 0.7	2	53.5 ± 1.0	2	61.0 ± 0.1	—		—		1
		*Pararoseomonas*	2	58.5 ± 0.2	2	64.3 ± 0.5	2	60.3 ± 0.7	—		—		—
		*Parasaccharibacter*	1	58.0	5	54.9 ± 0.1	—		—		—		3
		*Plastoroseomonas*	2	60.3 ± 0.7	2	62.9 ± 0.1	1	60.3	—		—		—
		*Pseudoroseomonas*	11	59.9 ± 0.4	4	62.7 ± 1.2	4	59.8 ± 0.6	—		—		—
		***Rhodopila*** ^32^	5	60.7 ± 0.4	5	58.8 ± 0.6	4	61.7 ± 0.9	—		1	69.0	1
		*Rhodovarius*	2	59.9 ± 0.3	2	56.9 ± 0.2	2	59.3 ± 0.8	—		—		1
		*Rhodovastum*	1	61.8	1	59.6	1	62.0	—		—		1
		*Roseicella*	4	60.3 ± 0.6	4	53.8 ± 0.3	4	61.2 ± 1.0	—		—		—
		*Roseococcus*	9	60.6 ± 0.4	10	55.5 ± 1.1	8	60.2 ± 0.5	—		—		3
		***Roseomonas***	33	59.8 ± 1.6	24	61.8 ± 2.1	21	59.9 ± 0.9	—		—		34
		*Rubritepida*	1	60.8	1	57.8	1	60.9	—		—		1
		*Sabulicella*	1	60.8	1	57.6	1	58.7	—		—		—
		*Saccharibacter*	3	58.8 ± 0.2	2	55.2 ± 0.1	3	60.5 ± 0.3	—		—		1
		*Sediminicoccus*	1	60.5	1	54.5	1	59.9	—		—		—
		*Siccirubricoccus*	4	60.5 ± 0.6	4	55.8 ± 1.1	4	61.4 ± 0.8	—		—		—
		*Swaminathania*	1	61.0	1	57.6	1	59.7	—		—		—
		*Swingsia*	1	57.7	1	56.0	1	60.9	—		—		—
		*Tanticharoenia*	1	58.9	1	57.0.8	1	61.0	—		1	81.6	—
	*Azospirillaceae*	***Azospirillum***	62	65.3 ± 1.6	31	55.7 ± 1.2	31	64.3 ± 1.5	—		—		30
		***Nitrospirillum***	8	66.0 ± 0.4	8	60.7 ± 0.2	3	66.7 ± 0.7	—		—		4
		***Skermanella***	6	66.6 ± 0.2	6	57.3 ± 1.1	6	61.4 ± 0.8	—		—		2
	*Rhodospirillaceae*	*Algihabitans*	1	67.5	1	58.0	1	68.3	—		—		—
		*Caenispirillum*	2	66.2 ± 0.4	2	59.1 ± 0.6	2	60.5 ± 0.9	—		—		—
		*Defluviicoccus*	1	62.7	1	57.2	1	62.0	—		1	64.1	—
		*Dongia*	4	67.5 ± 0.4	3	59.3 ± 0.8	4	60.7 ± 1.0	1	60.4	—		1
		*Ferruginivarius*	1	65.5	1	55.9	1	66.6	—		—		—
		***Hypericibacter***	2	68.0 ± 0.5	2	55.6 ± 1.5	2	63.3 ± 0.2	—		2	58.5 ± 0.3	2
		*Inquilinus*	5	66.1 ± 0.4	5	58.1 ± 0.8	5	62.8 ± 0.3	—		—		4
		***Magnetospirillum***	22	66.1 ± 2.6	12	57.2 ± 0.5	13	61.5 ± 1.0	—		—		13
		*Pararhodospirillum*	2	68.2 ± 0.1	2	55.8 ± 1.1	2	61.1 ± 1.7	—		—		4
		*Phaeovibrio*	1	67.5	1	53.8	—		—		—		—
		*Rhodospira*	2	67.6 ± 0.3	2	56.3 ± 1.3	2	61.7 ± 0.9	—		—		—
		***Rhodospirillum*** ^12,33–34^	3	67.2 ± 2.0	2	55.7 ± 0.1	3	63.0 ± 0.1	—		—		7
		*Roseospira*	4	67.0 ± 0.2	4	56.1 ± 1.1	4	61.8 ± 2.2	—		—		1
		*Roseospirillum*	1	64.0	1	54.2	1	63.3	—		—		1
		*Telmatospirillum*	3	68.0 ± 1.7	2	57.1 ± 1.1	2	63.1 ± 0.3	—		—		4
	*Rhodovibrionaceae*	***Limimonas***	1	64.1	1	54.5	1	59.7	—		—		1
		***Pelagibius***	3	68.5 ± 0.9	4	55.8 ± 0.4	4	64.0 ± 1.0	—		—		1
		***Tistlia***	1	67.6	1	55.3	1	65.2	—		1	64.4	1
	*Shumkoniaceae*	*Shumkonia*	1	67.0	2	54.0 ± 1.2	1	64.1	—		—		—
	*Stellaceae*	*Stella*	1	65.2	1	52.5	1	65.9	—		—		2
	*Thalassospiraceae*	*Magnetospira*	1	67.8	1	55.5	1	62.3	—		—		—
	*Incertae sedis*	***Enhydrobacter***	4	61.3 ± 8.5	2	63.8 ± 0.7	2	57.9 ± 0.4			—		1
*Sphingomonadales* ^e^	*Erythrobacteraceae*	*Erythrobacter*	64	61.2 ± 1.0	1	61.2	1	59.8	—		—		18
		***Novosphingobium***	141	60.6 ± 0.8	28	60.3 ± 2.2	25	58.1 ± 1.1	—		—		41
	*Sphingomonadaceae*	*Hephaestia*	3	61.2 ± 1.0	1	60.9	1	58.6	—		—		1
		***Sphingobium***	123	61.7 ± 0.7	2	61.3 ± 0.6	2	59.8 ± 0.4	—		—		44
		***Sphingomonas***	409	61.2 ± 1.0	77	62.1 ± 1.5	66	58.4 ± 1.3	—		—		157
		***Stakelama***	5	62.9 ± 0.9	1	62.2	1	60.4	—		—		2
	*Sphingosinicellaceae*	*Polymorphobacter*	8	63.0 ± 0.6	3	59.6 ± 0.1	3	57.9 ± 0.2	—		—		2
		*Thermaurantiacus*	1	63.7	1	56.0	1	69.3	—		—		—
	*Zymomonadaceae*	*Zymomonas* ^6,33‐34^	7	60.0 ± 0.2	9	59.6 ± 0.9	7	57.3 ± 0.7	—		—		10
Incertae sedis		*Thermopetrobacter*	1	71.5	1	52.7	1	59.9	—		—		—

*Note:* The genera listed contain at least the *shc* gene in one of their genomes (its absence in other genera is indicated in footnotes). The number of hits in the database of NCBI protein reference sequences and their average similarity (Sim, %) to the proteins of *Rhodopseudomonas palustris* TIE‐1 (GyrB, Shc, HpnH, and HpnP) and *Acetobacter pasteurianus* (HpnR) is listed. All detailed information with respect to the PSI‐BLAST searches is provided in Table S4. The taxonomic classification of APB is according to Hördt et al. (2020). The availability of strains at the DSMZ is indicated by the number of strains available (as per October 2023). All other genera not belonging to the orders *Hyphomicrobiales*, *Rhodobacterales*, *Rhodospirillales* and *Sphingomonadales* that do not contain a copy of the *shc* gene are: *Algimonas*, *Alkalicaulis*, *Amphiplicatus meriothermophilus*, *Anaplasma capra*, *Anaplasma*, *Aquarickettsia*, *Aquidulcibacter*, *Aquisalinus*, *Asticcacaulis*, *Bandiella*, *Brevundimonas*, *Deianiraea*, *Ehrlichia*, *Eilatimonas*, *Emcibacter*, *Euryhalocaulis*, *Finniella*, *Fokinia*, *Futiania*, *Gimibacter*, *Glycocaulis*, *Govania*, *Gromoviella*, *Hellea*, *Henriciella*, *Hepatobacter*, *Hirschia*, *Holospora*, *Hydrogenosomobacter*, *Hyphobacterium*, *Hyphococcus*, *Hyphomonas*, *Jidaibacter*, *Kordiimonas*, *Litorimonas*, *Luteithermobacter*, *Magnetaquicoccus*, *Magnetococcus*, *Magnetofaba*, *Maricaulis*, *Marinicauda*, *Marinicaulis*, *Megaera*, *Midichloria*, *Neoehrlichia*, *Neorickettsia*, *Nucleicultrix*, *Oceanibacterium*, *Oceanicaulis*, *Odyssella*, *Orientia*, *Paracaedibacter*, *Paremcibacter*, *Phycorickettsia*, *Phycosocius*, *Ponticaulis*, *Pseudaquidulcibacter*, *Pseudemcibacter*, *Pseudokordiimonas*, *Puniceispirillum*, *Rhodothalassium*, *Rickettsia*, *Robiginitomaculum*, *Sarmatiella*, *Sneabacter*, *Sneathiella*, *Temperatibacter*, *Terricaulis*, *Trichorickettsia*, *Viadribacter*, *Vitreimonas*, *Wolbachia*, *Woodsholea*.

Abbreviations: # = number of hits upon PSI BLAST searches, sim (%) = % similarity in protein sequence (±1 standard deviation).

^a^ Genera in bold typeface species have been examined in this study (see Table 1). For the underlined genera, species have been examined for the production of hopanoids and BHPDs in earlier studies. The numbers in superscript refer to these publications: (1) Vilchèze et al. 1994, (2) van Winden et al. (2012), (3) Schwartz‐Narbonne et al. (2020), (4) Neunlist and Rohmer (1985a), (5) Neunlist et al. (1988), (6) Flesch and Rohmer (1988), (7) Talbot et al. (2007a), (8) Kannenberg et al. (1995), (9) Bravo et al. (2001), (10) Komaniecka et al. (2014), (11) Elling et al. (2020), (12) Rohmer et al. 1984; (13) Talbot et al. (2007b), (14) Rashby et al. (2007), (15) Neunlist et al. (1985), (16) Lodha et al. (2015), (17) Zundel and Rohmer (1985), (18) Bisseret et al. 1985, (19) Renoux and Rohmer (1985), (20) Stampf et al. (1991), (21) Talbot et al. (2001), (22) Neunlist and Rohmer (1985b), (23) Srinivas et al. (2014), (24) Rohmer and Ourisson (1976a), (25) Herrmann et al. (1996), (26) Kogan et al. (1992), (27) Peiseler and Rohmer (1992), (28) Simonin et al. (1994), (29) Rohmer and Ourisson (1976b), (30) Rohmer and Ourisson (1986), (31) Hopmans et al. (2021), (32) Mayer et al. (2021), (33) Barrow and Chuck (1990), (34) Llopiz et al. (1992), (35) Schmidt et al. (1986), (36) Hermans et al. (1991).

^b^ Genera that do not contain a copy of the *shc* gene in this order are: *Aestuariivirga*, *Afifella*, *Ahrensia*, *Aliihoeflea*, *Allorhizobium*, *Alsobacter*, *Aminobacter*, *Amorphus*, *Ancyclobacter*, *Angulomicrobium*, *Antarcticirhabdus*, *Aquabacter*, *Aquamicrobium*, *Aquibium*, *Aquibium*, *Arsenicitalea*, *Aurantimonas*, *Azorhizobium*, *Bauldia*, *Blastochloris*, *Bosea*, *Breoghania*, *Brucella*, *Camelimonas*, *Chelativorans*, *Chenggangzhangella*, *Chthonobacter*, *Ciceribacter*, *Cohaesibacter*, *Consotaella*, *Corticibacterium*, *Cucumibacter*, *Devosia*, *Dichotomicrobium*, *Ectorhizobium*, *Endobacterium*, *Falsochrobactrum*, *Fererhizobium*, *Filomicrobium*, *Flavimaribacter*, *Fodinicurvata*, *Fulvimarina*, *Gellertiella*, *Georhizobium*, *Hartmannibacter*, *Hoeflea*, *Hongsoonwoonella*, *Jiella*, *Kaistia*, *Labrenzia*, *Lentilitoribacter*, *Liberibacter*, *Limibacillus*, *Limoniibacter*, *Lutibaculum*, *Mangrovicella*, *Mariluticola*, *Maritalea*, *Martelella*, *Mesorhizobium*, *Methylobrevis*, *Methyloceanibacter*, *Methyloligella*, *Methylopila*, *Microbaculum*, *Mongoliimonas*, *Mycoplana*, *Neorhizobium*, *Nitratireductor*, *Nordella*, *Notoacmeibacter*, *Oceaniradius*, *Ochrobactrum*, *Oharaeibacter*, *Oricola*, *Oryzibacter*, *Oryzicola*, *Paenochrobactrum*, *Pannonibacter*, *Paradevosia*, *Paramesorhizobium*, *Pararhizobium*, *Parvibaculum*, *Pelagibacterium*, *Peteryoungia*, *Phreatobacter*, *Phyllobacterium*, *Pinisolibacter*, *Pleomorphomonas*, *Propylenella*, *Prosthecodimorpha*, *Prosthecomicrobium*, *Pseudaminobacter*, *Pseudochelatococcus*, *Pseudochrobactrum*, *Pseudohoeflea*, *Pseudorhizobium*, *Pseudorhodoplanes*, *Pseudovibrio*, *Pseudoxanthobacter*, *Pyruvatibacter*, *Rhabdaerophilum*, *Rhizobium*, *Rhodobium*, *Rhodovibrio*, *Roseibium*, *Roseitalea*, *Salaquimonas*, *Saliniramus*, *Segnochrobactrum*, *Shinella*, *Siculibacillus*, *Stappia*, *Tepidamorphus*, *Tepidicaulis*, *Tianweitania*, *Tokpelaia*, *Undibacter*, *Xanthobacter*, *Xaviernesmea*, *Youhaiella*, *Zhengella*.

^c^ Genera that do not contain a copy of the *shc* gene in this order are: *Abyssibius*, *Acidimangrovimonas*, *Actibacterium*, *Aestuariicoccus*, *Agaricicola*, *Albibacillus*, *Albidovulum*, *Albimonas*, *Algicella*, *Aliiroseovarius*, *Aliiruegeria*, *Aliishimia*, *Alisedimentitalea*, *Alkalilacustris*, *Allgaiera*, *Allosediminivita*, *Alloyangia*, *Alterinioella*, *Amaricoccus*, *Amylibacter*, *Anianabacter*, *Antarcticimicrobium*, *Antarctobacter*, *Aquicoccus*, *Aquimixticola*, *Arenibacterium*, *Ascidiaceihabitans*, *Boseongicola*, *Brevirhabdus*, *Celeribacter*, *Chachezhania*, *Citreicella*, *Citreimonas*, *Cochlodiniinecator*, *Cognatishimia*, *Cribrihabitans*, *Cypionkella*, *Defluviimonas*, *Dinoroseobacter*, *Donghicola*, *Epibacterium*, *Falsigemmobacter*, *Falsihalocynthiibacter*, *Falsirhodobacter*, *Falsiruegeria*, *Fertoeibacter*, *Flavimaricola*, *Fluviibacterium*, *Frigidibacter*, *Fuscibacter*, *Fuscovulum*, *Gemmobacter*, *Gymnodinialimonas*, *Gymnodinialimonas*, *Haematobacter*, *Halocynthiibacter*, *Halovulum*, *Hasllibacter*, *Heliomarina*, *Histidinibacterium*, *Jannaschia*, *Jhaorihella*, *Kandeliimicrobium*, *Kangsaoukella*, *Ketogulonicigenium*, *Leisingera*, *Lentibacter*, *Limimaricola*, *Litoreibacter*, *Litorivita*, *Loktanella*, *Lutimaribacter*, *Maliponia*, *Mameliella*, *Mangrovicoccus*, *Maribius*, *Marimonas*, *Marinibacterium*, *Marinovum*, *Maritimibacter*, *Marivita*, *Marivivens*, *Meinhardsimonia*, *Meridianimarinicoccus*, *Mesobacterium*, *Mesobaculum*, *Monaibacterium*, *Muriiphilus*, *Natronohydrobacter*, *Neomegalonema*, *Nereida*, *Nioella*, *Oceanibium*, *Oceanicella*, *Oceanicola*, *Oceaniglobus*, *Oceaniovalibus*, *Oceanomicrobium*, *Octadecabacter*, *Pacificibacter*, *Pacificitalea*, *Paenihalocynthiibacter*, *Paenimaribius*, *Paenirhodobacter*, *Palleronia*, *Paracoccus*, *Pararhodobacter*, *Parasedimentitalea*, *Paroceanicella*, *Pelagicola*, *Pelagimonas*, *Pelagivirga*, *Pelagovum*, *Phaeobacter*, *Phaeovulum*, *Phycocomes*, *Pikeienuella*, *Planktomarina*, *Planktotalea*, *Polymorphum*, *Pontibaca*, *Pontibrevibacter*, *Ponticoccus*, *Pontivivens*, *Poseidonocella*, *Primorskyibacter*, *Profundibacterium*, *Pseudaestuariivita*, *Pseudodonghicola*, *Pseudogemmobacter*, *Pseudohalocynthiibacter*, *Pseudooceanicola*, *Pseudooctadecabacter*, *Pseudophaeobacter*, *Pseudopuniceibacterium*, *Pseudorhodobacter*, *Pseudoroseicyclus*, *Pseudoruegeria*, *Pseudosulfitobacter*, *Pseudotabrizicola*, *Pseudothioclava*, *Psychromarinibacter*, *Pukyongiella*, *Puniceibacterium*, *Qingshengfaniella*, *Rhabdonatronobacter*, *Rhodobaca*, *Rhodobacter*, *Rhodobaculum*, *Rhodophyticola*, *Rhodosalinus*, *Roseibaca*, *Roseibacterium*, *Roseicitreum*, *Roseicyclus*, *Roseinatronobacter*, *Roseisalinus*, *Roseitranquillus*, *Roseivivax*, *Roseobacter*, *Roseovarius*, *Rubellimicrobium*, *Rubricella*, *Rubrimonas*, *Ruegeria*, *Sagittula*, *Salibaculum*, *Salinihabitans*, *Salipiger*, *Sedimentimonas*, *Sedimentitalea*, *Sediminimonas*, *Seohaeicola*, *Shimia*, *Silicimonas*, *Sinirhodobacter*, *Sinisalibacter*, *Solirhodobacter*, *Stagnihabitans*, *Sulfitobacter*, *Szabonella*, *Tabrizicola*, *Tateyamaria*, *Thalassobacter*, *Thalassobium*, *Thalassobius*, *Thalassococcus*, *Thalassorhabdomicrobium*, *Thalassovita*, *Thetidibacter*, *Thioclava*, *Thiosulfatihalobacter*, *Tranquillimonas*, *Tritonibacter*, *Tropicimonas*, *Vannielia*, *Wenxinia*, *Xinfangfangia*, *Yoonia*, *Youngimonas*, *Zongyanglinia*.

^d^ Genera that do not contain a copy of the *shc* gene in this order are: *Aestuariispira*, *Aliidongia*, *Arboricoccus*, *Arenibaculum*, Ca. *Endolissoclinum*, *Curvivirga*, *Denitrobaculum*, *Elioraea*, *Elstera*, *Falsiroseomonas*, *Ferrovibrio*, *Geminicoccus*, *Haematospirillum*, *Humitalea*, *Hwanghaeella*, *Indioceanicola*, *Insolitispirillum*, *Kiloniella*, *Magnetovibrio*, *Marivibrio*, *Neoroseomonas*, *Nisaea*, *Niveispirillum*, *Novispirillum*, *Oceanibaculum*, *Oleisolibacter*, *Oleomonas*, *Pacificispira*, *Paeniroseomonas*, *Terasakiella*, *Thalassobaculum*, *Thalassospira*, *Tistrella*, *Varunaivibrio*, *Zavarzinia*.

^e^ Genera that do not contain a copy of the *shc* gene in this order are: *Actirhodobacter*, *Allopontixanthobacter*, *Allosphingosinicella*, *Alteraurantiacibacter*, *Altererythrobacter*, *Altericroceibacterium*, *Alteripontixanthobacter*, *Alteriqipengyuania*, *Aquisediminimonas*, *Aurantiacibacter*, *Blastomonas*, *Caenibius*, *Citromicrobium*, *Croceibacterium*, *Croceicoccus*, *Glacieibacterium*, *Novosphingopyxis*, *Pacificimonas*, *Parapontixanthobacter*, *Parasphingopyxis*, *Parasphingorhabdus*, *Paraurantiacibacter*, *Parerythrobacter*, *Parerythrobacter*, *Parerythrobacter*, *Parerythrobacter*, *Pedomonas*, *Pelagerythrobacter*, *Pontixanthobacter*, *Porphyrobacter*, *Pseudomonadota*, *Pseudopontixanthobacter*, *Qipengyuania*, *Rhizorhabdus*, *Rhizorhapis*, *Sandaracinobacter*, *Sandaracinobacteroides*, *Sandarakinorhabdus*, *Sphingomicrobium*, *Sphingopyxis*, *Sphingorhabdus*, *Sphingosinicella*, *Sphingosinithalassobacter*, *Tardibacter*, *Tsuneonella*.

Table 2 summarizes the results of the PSI‐BLAST searches for the Shc (= HpnF) protein of *R. palustris* TIE‐1 and represents an overview of the genera that hold one or more species that contain the *shc* gene in their genomes. In some species, two proteins annotated as Shc were detected (e.g., *Zymomonas mobilis* ), but the second is more distantly (< 40% similarity at the protein level) related to Shc of *R. palustris* , and the corresponding *shc* gene is not part of the BHPD gene cluster (see below). This “second” group of Shc proteins occurs in only a limited group of species and forms a distinct, separate clade in the phylogenetic tree of all Shc sequences (data not shown) and was not taken into consideration in further data handling. Overall, 1409 Shc sequences were obtained (i.e., ca. 23% of all ca. 6500 genomes encoding unique GyrB sequences), which are unevenly distributed over the orders of the APBs: *Hyphomicrobiales* (791 hits; ca. 30%), *Rhodobacterales* (4; < 1%), *Rhodospirillales* (475; ca. 70%), *Sphingomonadales* (123; ca. 11%), and other orders (16; ca. 2%) (Table 2, Table S4). This can also be considered at the level of genera. For the *Hyphomicrobiales*, 46 out of 171 genera (27%) have at least one genome with a copy of the *shc* gene. For the *Rhodobacterales*, *Rhodospirillales*, *Sphingomonadales*, and other orders, this amounts to 3 out of 203 (1.5%), 73 out of 106 (69%), 9 out of 51 (18%), and 7 out of 78 (9%), respectively. Evidently, the genetic capability to be able to cyclize squalene is unevenly distributed over the various orders of APBs, that is, it predominantly occurs in the *Rhodospirillales* and, to a lesser extent, in the *Hyphomicrobiales*, and is much more restricted in the other orders. Hence, based on the presence of *shc* detected in our analyses, the ability for hopanoid biosynthesis occurs much more limited in APB than commonly assumed. This is further underscored by the fact that in many genera just a few species possess the *shc* gene (cf. number of GyrB and Shc proteins per genus; Table 2); for example, all *Bradyrhizobium* genomes contain the *shc* gene, whilst only 2 out of the 123 *Sphingobium* genomes contain the *shc* gene.

To assess the genomic potential for BHPD production, we also performed PSI‐BLAST searches with the HpnH protein of *R. palustris* TIE‐1, which catalyzes the addition of the 5′‐deoxyadenosyl radical to diploptene (Bradley et al. 2010; Sato et al. 2020, 2021), the first and crucial step in the biosynthesis of C_35_ BHPDs (Figure 1a). The *hpnH* gene was detected in almost all APB genomes that encode *shc*, with only a few exceptions (Table 2, Table S4), in line with earlier work for a much more limited group of the APB (Tookmanian et al. 2021). This suggests that almost all APB that possess the *shc* gene also possess the *hpnH* gene and, thus, also have the potential to produce BHPDs.

We further searched for the proteins catalyzing ring‐A methylation of C_30_ hopanoids and BHPDs encoded by the genomes of the APB. These are the HpnP and HpnR proteins, responsible for the methylation at C‐2 and C‐3, respectively. For HpnP, PSI‐BLAST searches with the well‐characterized HpnP protein of *R. palustris* TIE‐1 were used (Welander et al. 2010; Table S2). For this search, the cut‐off had to be quite stringent because other radical SAM proteins have substantial similarity (see Elling et al. 2020 and Hoshino et al. 2023 for detailed discussion). Only HpnP sequences with a sequence similarity of ca. 55% or more (after two PSI‐BLAST iterations) were considered to reflect enzymes enabling the methylation at C‐2. All other B12‐binding domain‐containing radical SAM proteins had a sequence similarity < 34% and were part of other phylogenetic clades. The *hpnP* gene is much less widely distributed in the APB than the *shc* and *hpnH* genes (Table 2, Table S4); it is limited to genera of the *Hyphomicrobiales* with two exceptions: the genus *Rhodovulum* of the *Rhodobacteriales* (one genome) and the genus *Dongia* of the *Rhodospirillales* (one out of four genomes). Within the families of the *Hyphomicrobiales* that contain species with the *shc* and *hpnH* genes, *hpnP* occurrence is much more common; it occurs in the families *Beijerinckiaceae*, *Nitrobacteraceae*, *Hyphomicrobiaceae*, *Licheni‐habitantaceae*, *Methylobacteriaceae*, *Methylocystaceae*, and *Parvibaculaceaea* (Table 2). In two of these families (i.e., the *Beijerinckiaceae* and *Lichenihabitantaceae*), all genera that have at least one species possessing *shc* in their genomes also possess *hpnP*. In the other families, there are genera that lack the *hpnP* gene even though they possess the *shc* and *hpnH* genes in their genomes (Table 2, Table S4).

For the PSI‐BLAST searches for HpnR (Welander and Summons 2012), we used HpnR of *Acetobacter pasteurianus* (Table S2) because the genome of our “reference BHPD‐producing” species *R. palustris* lacks the *hpnR* gene and *A. pasteurianus* is well known for its production of 3‐methyl hopanoids (Zundel and Rohmer 1985). The genetic capacity to produce 3‐methyl hopanoids in the APB is mainly restricted to the family *Acetobacteraceae* of the order *Rhodospirillales*; in 10 out of 51 families with species possessing genomes containing the *shc* and *hpnH* genes, HpnR was encoded by their genomes (Table 2, Table S4). In addition, this protein was encoded by genomes of the genera *Defluviicoccus* (1 genome) and *Hypericibacter* (2 genomes) in the family *Rhodospirillaceae* belonging to the order *Rhodospirillales* and by genomes of the genera *Methylobacterium*, *Microvirga*, *Reyranella*, and *Tistlia* of the genus *Hyphomicrobiales*. Remarkably, in the genus *Methylobacterium* in 3 of the almost 100 available genomes, *hpnR* is present in addition to *shc*, *hpnH*, and *hpnP*. This suggests that these 3 species may possess the capacity to produce 2,3‐dimethyl hopanoids, as has been previously demonstrated for an acidobacterium (Sinninghe Damsté et al. 2017).

In summary, our PSI‐BLAST searches revealed that BHPD biosynthesis in APB is more limited than commonly assumed. This is even more pronounced for the occurrence of the *hpnP* and *hpnR* genes, indicating that the potential to biosynthesize ring‐A methylated BHPDs by present‐day APBs is quite restricted.

### 3.2 Design of the Cultivation Approach

**FIGURE 2:**
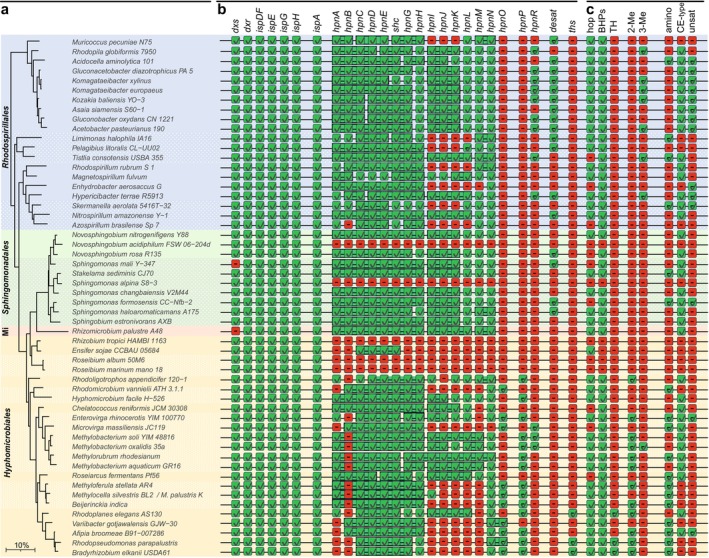
The genomic potential of biosynthesis and actual production of C_30_ hopanoids, tetrahymanol, and BHPDs in 54 species of APB. (a) 16S rRNA phylogenetic tree of the studied species showing their subdivision into the four different orders (Mi stands for *Micropepsales*). The different families of the orders (see Table 1) are indicated by subtle changes in the background color. As outgroup for the construction of the 16S rRNA gene of the gammaproteobacterium *Methylococcus capsulatus* was used (not included in the tree). The accession numbers of all sequences used are listed in Table S3. (b) The presence/absence of genes encoding proteins catalyzing the production of BHPDs. The first group of genes encode the MEP pathway. *IspA* encodes farnesyl diphosphate synthase. The following group of genes encodes the various proteins involved in BHPD synthesis starting with farnesyl diphosphate (Figure 1a). They often occur in a BGC (Figure 1b) and instances where the genes occur directly after each other in the genome in the designated order are indicated by an association of the tick signs. Often, however, genes of the *hpn* cluster not indicated as such still occur in the very close vicinity of other *hpn* genes. The next two genes, *hpnP* and *hpnR*, encode SAM radical proteins responsible for methylation of C_30_ hopanoids, tetrahymanol, and BHPDs at position C‐2 and C‐3, respectively. *Desat* is a gene annotated as a “sterol desaturase” and potentially involved in the formation of double bonds in BHPDs (see text). *Ths* is the gene encoding tetrahymanol synthase. When the green boxes with the tick sign (meaning “gene is present”) are connected the *hpn* genes are adjacent to each other and follow their defined A‐R order. If this is not the case, *hpn* genes still may be closely associated forming the *hpn*‐BGC (see Figure 1b for examples). All detailed information with respect to the PSI‐BLAST searches is provided in Table S5. (c) The presence/absence of C_30_ hopanoids (hop), BHPDs, tetrahymanol (TH), their 2‐ and 3‐methylated derivatives (2‐Me and 3‐Me, respectively), and specific classes of BHPDs, that is, those with an amino moiety at C‐35 (amino), those belonging to the group of BHPD CEs, including intermediates (CE‐type), and unsaturated BHPDs (unsat).

**FIGURE 3:**
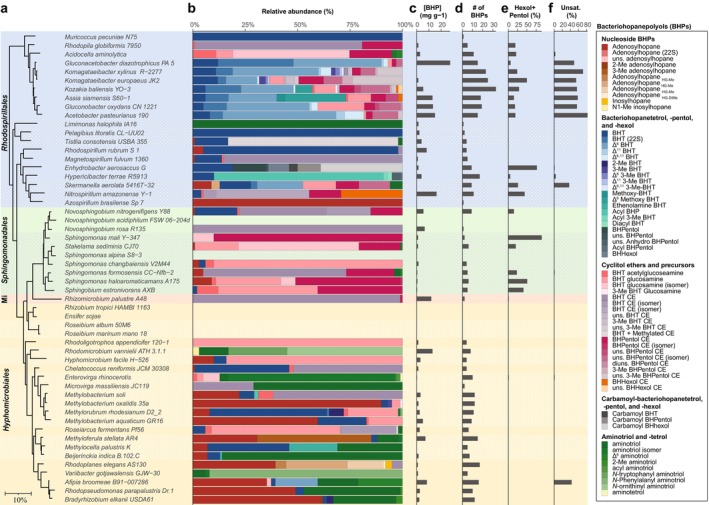
Distribution and abundance of BHPDs in 54 species of APB. (a) 16S rRNA phylogenetic tree of the studied bacteria showing their subdivision into the four different orders (see for details the caption of Figure 2). (b) Relative abundance of the BHPDs detected in the bacterial cultures. Minor BHPDs (i.e., BHPDs that did not exceed 0.5% in any culture) were left out. Color codes refer to the extended caption at the right‐hand side and are ordered to reveal related groups of BHPDs. Roman numerals refer to the structures shown in Figure 5. The relative abundances of the BHPDs are also provided in Table S6. (c) Absolute abundance of BHPDs (in mg g^−1^ dried cells excluding nucleosides) as determined by the Rohmer degradation method (see Methods). (d) Total numbers of individual BHPDs identified in each culture (including minor and trace constituents). (e) The relative abundance of BHpentol and ‐hexol derivatives as determined with the Rohmer degradation method. (f) The relative abundance of BHPDs containing one or more double bonds in the ring system.

Based on these PSI‐BLAST searches and previous investigations on hopanoids and BHPDs in APB cultures, the availability of strains of interest in the DSMZ culture collection, and the availability of sequenced genomes of these strains in the NCBI database, 52 different strains were selected (see Table 1 for strain designations; these will not be repeated in the main text) and cultivated to further expand our knowledge of BHPD synthesis by APB and to study the relationship between the presence of BHPD biosynthetic genes and their actual production (i.e., genotype vs. phenotype). This set was complemented with one species that had previously been studied, that is, *Komagataeibacter xylinus* (Hopmans et al. 2021), and *Methylocella palustris* obtained as a gift, leading to a total of 54 strains analyzed in this study. The selected strains cover (i) genera that contain *hpnP* and *hpnR* in their genomes and hence are potentially capable of producing 2‐ and 3‐methyl BHPDs but have not previously been tested to produce them, (ii) representatives of families/genera that do seem to be able to produce BHPDs based on their genomic composition but have not been tested for BHPD production, (iii) two genera (i.e., *Novosphingobium* and *Sphingomonas*) for which the genomic capacity to produce BHPD varies at the species level, and (iv) three *Methylobacterium* species that vary in the presence of the *hpnP* and *hpnR* genes. The selected 54 strains cover the main families of the three orders of APB and the taxonomical classification of the studied species is listed in Table 1 and graphically represented in Figures 2a and 3a. Five of the 54 strains studied had a genomic composition (i.e., they lacked the *shc* gene; Table S5) indicating that they would not be capable of BHPD biosynthesis and were used as negative controls. The selected strains represent a set of species that allow for genotypic versus phenotypic comparison of hopanoid production in APB; however, due to the limited availability of cultured strains, the species selection underrepresents certain natural biomes (e.g., only three marine species were included, whereas the oceans cover 70% of the surface of the Earth).

The genomes of all 54 strains (or in a few cases phylogenetically very closely related strains; see footnotes in Table 1) were studied in detail for all known genes involved in BHPD biosynthesis (Table S2). In addition, these strains were also cultivated and analyzed for the production of C_30_ hopanoids and BHPDs. To study the BHPD composition, two approaches were followed. First, the extracts were directly analyzed by our newly developed UHPLC‐HRMS^n^ technique, which allows for the analysis of non‐derivatized BHP composites (Hopmans et al. 2021), in contrast to the analysis of BHPDs after acetylation (Talbot et al. 2001, 2007a). This new analytical technique enables the separation and identification of a broad range of BHPDs, including relatively simple BHPDs to nitrogen‐containing BHPDs and complex composite BHPDs. Second, we used a chemical degradation technique using periodic acid/sodium borohydride to convert BHPDs into GC‐amenable hopanoid alcohols (hopanols) (Rohmer et al. 1984). For this so‐called Rohmer degradation, lyophilized cells were directly treated. By applying this method in the study of *Acidobacteria*, Sinninghe Damsté et al. (2017) previously demonstrated that this may aid in the detection of a fraction of “hidden” BHPDs, as observed in the APB *Acetobacter xylinum* (Herrmann et al. 1996). BHPDs also occur covalently bound in hard‐to‐extract large molecules such as Lipid A (Komaniecka et al. 2014). Therefore, the Rohmer degradation on intact cells provides an additional check for the presence and structural diversity of the hopanoid inventory of a species. A drawback of this method is that not all BHPDs can be detected in this way; two vicinal hydroxy groups are required for the cleavage of the side chain of the BHPD, which is not always the case, as exemplified by adenosylhopanes (Neunlist et al. 1988). The presence of C_30_ hopanoids and the related triterpenoid, tetrahymanol, was also studied in the fractions obtained by the Rohmer degradation followed by GC–MS analysis.

### 3.3 Identification of BHPD Genes/Proteins of Selected APB Strains

For the 54 selected strains (Table 1), the presence/absence of all known genes encoding the biosynthetic steps in the BHPD pathways (Figure 1a, Table S2) in their genomes were mapped through a search for their proteins encoded by these genes in the NCBI RefSeq database (Table S5). These data are summarized in Figure 2b.

The genomes of all strains possess the genes (*dxs*, *dxr*, *ispD*, *ispE*, *ispF*, *ispG*, *ispH*) encoding the proteins of the MEP pathway of isoprenoid biosynthesis (Zhao et al. 2013) (Figure 2b). In all but two genomes, *ispD* and *ispF* are combined into an *ispDF* gene. Two species (i.e., *Sphingomonas mali* and *Rhizomicrobium palustre* ) are lacking *dxs*. This has been previously observed for some BHPD‐producing acidobacteria (Sinninghe Damsté et al. 2017) and may be compensated for by the presence of a punt‐mutated *aceE* (Sauret‐Güeto et al. 2006), which was indeed detected in *S. mali* , but not in *R. palustre* . All genomes also contained the gene encoding farnesyl diphosphate synthase (*ispA*), providing the strains the genetic capacity to produce the isoprenoid C_15_ building block used to produce squalene, an important intermediate for BHPD synthesis.

With respect to the 6 key genes encoding proteins for the BHPD biosynthetic pathway (i.e., *hpnC*, *hpnD*, *hpnE*, *shc*, *hpnG*, and *hpnH*; Figure 1a), five species (i.e., *Rhizobium tropici* , *Roseibium album*, *R. marinum* , *Novosphingobium acidiphilum* , and *Sphingomonas alpina*; the negative control strains) lacked all these genes (Figure 2b, Table S5). *Ensifer sojae* lacked *hpnG* and *hpnH* (Figure 2b), suggesting it may be capable of C_30_ hopanoid but not of BHPD production. All other investigated strains contained these 6 key genes of the BHPD biosynthetic pathway in their genomes. Most of these 6 key genes were co‐localized, presumably forming a so‐called biosynthetic gene cluster (BGC; e.g., Medema et al. 2015). Examples of this co‐localization are shown in Figure 1b and are also denoted in Figure 2b (see the caption for an explanation).

The three genes (i.e., *hpnI*, *hpnK*, and *hpnJ*) encoding the proteins for the stepwise production of bacteriohopanetetrol (BHT) acetylglucosamine, BHT glucosamine, and BHT cyclitol ether, respectively (Figure 1a), are either all present or all absent (Figure 2b, Table S5). The presence of these genes is not related to the taxonomic/phylogenetic position of the species. There are two exceptions: *Methylocella* spp. only possess *hpnI* of these three genes, whilst *Rhodoligotrophos appendicifer* lacks *hpnJ*. In the genome architecture, the *hpnI*, *hpnK*, and *hpnJ* genes are closely associated (Figure 2b) but often do not follow the *hpnH* gene, as would be expected on the basis of the order in the nomenclature of the *hpn* genes. However, in most cases, they are often still part of a large *hpn* BGC (e.g., Figure 1b). The occurrence of *hpnO* encoding the protein responsible for the production of aminobacteriohopanetriol (Welander et al. 2012) is restricted to the investigated species of the *Hyphomicrobiales* (Figure 2b, Table S5) but occurs in only 45% of the species predicted to produce BHPDs. It typically is not part of the *hpn* BGC. As would be expected, the *hpnN* gene, encoding an integral membrane protein responsible for the transportation of BHPDs to the outer membrane (Doughty et al. 2011; Kumar et al. 2017), is present in all genomes of predicted BHPD‐producing APB (Figure 2b, Table S5). The exact function of the proteins encoded by the *hpnA* (BHPD‐associated sugar epimerase), *hpnB* (BHPD‐associated glycosyl transferase), *hpnL*, and *hpnM* remains elusive (Perzl et al. 1998; Schmerk et al. 2015). All of them are absent in the genomes that do not possess *hpnH* (and hence likely do not produce BHPDs), but they are not always present in species that possess a *hpn* BGC in their genomes; *hpnA* is absent in 6 of the 48 APB genomes containing *hpnH*, while *hpnB*, *hpnL*, and *hpnM* are absent in 13 (not all in the same species) of these 48 genomes (Figure 2b, Table S5). In most cases, these genes are part of the (partially fractured) BGC (Figures 1b and 2b). This suggests that they are likely involved in BHPD biosynthesis, but not in essential steps.

With respect to the genes responsible for the ring‐A methylation of hopanoids, *hpnP* was only detected in the genomes of 14 species, all falling in the order *Hyphomicrobiales* (Figure 2b, Table S5), in line with recent findings of Hoshino et al. (2023). The occurrence of the *hpnR* gene was restricted to 3 species of the *Hyphomicrobiales* and 9 of the order *Rhodospirillales* (Figure 2b, Table S5). The genomes of two out of the 54 selected strains contained both the *hpnP* and *hpnR* genes. These were *Methylobacterium* spp. (i.e., *M. oxalidis* and *M. soli* ; Figure 2, Table S5). The genes encoding the proteins for ring‐A methylation (i.e., *hpnP* and *hpnR*) were usually not co‐localized with other BHPD genes in the APB genomes analyzed (Figures 1b and 2b).

### 3.4 C_30_ Hopanoid and BHPD Composition as Determined by Rohmer Degradation

The summed concentrations of C_30_ hopanoids (including ring‐A methyl derivatives) and tetrahymanol and 2‐methyltetrahymanol ranged widely from 0.02 to 7.1 mg g^−1^ biomass (Table 3). In 22 of the 54 examined species, C_30_ hopanoids were not detected. These included the 5 species lacking *shc* in their genome (i.e., *Rhizobium tropici* , *Roseibium album*, *R. marina* , *Novosphingobium acidiphilum* , *Sphingomonas alpina*), but also a lot of other species that did produce BHPDs (see below). The hopanoids detected were comprised of hop‐22(29)‐ene (diploptene), hop‐17(21)‐ene, diplopterol, and their 2‐methyl derivatives, which occurred only in species of the order *Hyphomicrobiales*, but not in any of the other orders examined. Remarkably, 2‐methyldiplopterol was more abundant than diplopterol itself in 5 out of the 9 species examined, resulting in a high degree of methylation (7%–97%; Table 4). Tetrahymanol was present in *Bradyrhizobium elkanii* , *Rhodoplanes elegans* , and *Rhodopseudomonas parapalustris* (Figure 2b), the only three species that contained the gene encoding tetrahymanol synthase (*ths*; Banta et al. 2015) (Figure 2b). This confirms earlier reports on its occurrence in *R. palustris* (Kleemann et al. 1990) and five *Rhodoplanes* species (Lodha et al. 2015). 2‐Methyl tetrahymanol was also detected in two of these species in considerable amounts (Table 4), and *R. parapalustris* also contained 20‐methyl‐tetrahymanol, a triterpenoid previously identified in the phylogenetically closely related *Bradyrhizobium japonica* (Bravo et al. 2001). Eickhoff et al. (2013) identified tetrahymanol containing two additional methyl groups at C‐2 and C‐20 in *R. palustris* , which was not detected in the strains investigated here.

**TABLE 3:** Concentrations of C_30_ hopanoids, tetrahymanol and summed BHPDs (as determined with the Rohmer method) in the various species of APB examined in this study.

Order	Family	Species–strain number	DSM culture number	Concentrations (mg/g dry weight)					22S/(22S + 22R) (%)	# Of BHPDs	% Unsaturated BHPDs	% Pentol plus hexol
				Hop‐22(29)‐ene	Hop‐21‐ene	Diploterol	Tetrahymanol	ΣBHPDs				
*Rhodospirillales*	*Acetobacteraceae*	*Muricoccus pecuniae* N75	25622^T^	—	—	—	—	0.1	—	1	0	0
		*Rhodopila globiformis* 7950	161^T^	0.12	—	—	—	1.3	—	5	0	19
		*Acidocella aminolytica* 101	11237^T^	0.11	—	—	—	2.7	—	11	3	21
		*Gluconacetobacter diazotrophicus* PA 5	5601^T^	—	—	—	—	27.3	79	15	49	6
		*Komagataeibacter xylinus* R‐2277	n.a.	n.d.	n.d.	n.d.	n.d.	n.d.	n.d	23	71	24
		*Komagataeibacter europaeus* JK2	13110	—	—	0.12	—	1.3	7	25	54	50
		*Kozakia baliensis* YO‐3	14400^T^	—	—	0.14	—	5.3	15	33	56	29
		*Asaia siamensis* S60‐1	15972^T^	0.07	0.08	0.88	—	12.9	14	18	58	15
		*Gluconobacter oxydans* CN 1221	2003	—	—	0.87	—	13.3	27	19	56	18
		*Acetobacter pasteurianus* 190	3509^T^	—	—	1.7	—	14.9	25	15	82	19
	*Rhodovibrionaceae*	*Limimonas halophila* IA16	25584^T^	—	—	0.05	—	1.4	—	1	0	0
		*Pelagibius litoralis* CL‐UU02	21314^T^	—	—	—	—	2.2	—	2	0	0
		*Tistlia consotensis* USBA 355	21585^T^	—	—	—	—	3.9	—	4	0	0
	*Rhodospirillaceae*	*Rhodospirillum rubrum* S 1	467^T^	0.38	—	—	—	8.1	—	4	0	0
		*Magnetospirillum fulvum* 1360	113^T^	—	—	—	—	0.9	—	5	0	0
	*Reyranellaceae*	*Enhydrobacter aerosaccus* G	8914^T^	—	—	—	—	1.4	—	9	1	77
	*Rhodospirillaceae*	*Hypericibacter terrae* R5913	109816^T^	0.27	0.04	—	—	3.9	—	17	4	5
	*Azospirillaceae*	*Skermanella aerolata* 5416 T‐32	18479^T^	—	—	—	—	—	—	9	37	26
		*Nitrospirillum amazonense* Y‐1	2787^T^	0.20	—	—	—	16.2	—	11	0	44
		*Azospirillum brasilense* Sp 7	1690^T^	—	—	—	—	—	—	1	0	0
*Sphingomonadales*	*Erythrobacteraceae*	*Novosphingobium nitrogenifigens* Y88	19370^T^	0.51	0.03	0.06	—	5.5	—	9	0	15
		*Novosphingobium acidiphilum* FSW 06‐204d	19966	—	—	—	—	—	—	0	n.a.	n.a.
		*Novosphingobium rosa* R13*5*	7285^T^	—	—	—	—	6.5	—	1	0	0
	*Sphingomonadaceae*	*Sphingomonas mali* Y‐347	10565^T^	—	—	—	—	1.1	—	2	0	90
		*Stakelama sediminis* CJ70	27203^T^	2.1	0.07	—	—	1.0	—	6	0	20
		*Sphingomonas alpina* S8‐3	22537^T^	—	—	—	—	—	—	0	n.a.	n.a.
		*Sphingomonas changbaiensis* V2M44	25652^T^	0.34	—	—	—	1.4	—	4	0	0
		*Sphingomonas formosensis* CC‐Nfb‐2	24164^T^	—	—	—	—	0.5	—	5	1	23
		*Sphingomonas haloaromaticamans* A175	13477^T^	0.03	—	—	—	0.6	—	6	0	51
		*Sphingobium estronivorans* AXB	102173^T^	—	—	—	—	0.4	—	5	0	41
*Micropepsales*	*Micropepsaceae*	*Rhizomicrobium palustre* A48	19867^T^	—	—	—	—	11.9	—	2	0	1
*Hyphomicrobiales*	*Rhizobiaceae*	*Rhizobium tropici* HAMBI 1163	11418^T^	—	—	—	—	—	—	0	n.a.	n.a.
		*Ensifer sojae* CCBAU 05684	26426^T^	—	—	—	—	—	—	0	n.a.	n.a.
	*Stappiaceae*	*Roseibium album* 50M6	18320^T^	—	—	—	—	—	—	0	n.a.	n.a.
		*Roseibium marinum* mano 18	17023^T^	—	—	—	—	—	—	0	n.a.	n.a.
	*Parvibaculaceae*	*Rhodoligotrophos appendicifer* 120‐1	23582^T^	—	—	2.8	—	1.3	—	4	0	0
	*Hyphomicrobiaceae*	*Rhodomicrobium vannielii* ATH 3.1.1	162^T^	0.48	—	—	—	12.9	—	8	0	0
		*Hyphomicrobium facile* H‐526	1565^T^	—	—	0.02	—	3.0	—	4	0	0
	*Chelatococcaceae*	*Chelatococcus reniformis* JCM 30308	105737^T^	—	—	—	—	1.3	—	4	0	0
	*Methylobacteriaceae*	*Enterovirga rhinocerotis* YIM 100770	25,903^T^	0.05	0.06	0.46	—	0.3	—	10	1	1
		*Microvirga massiliensis* JC119	26813^T^	—	—	—	—	—	—	2	0	0
		*Methylobacterium soli* YIM 48816	21955^T^	0.13	0.09	1.4	—	3.1	—	12	0	0
		*Methylobacterium oxalidis* 35a	24028^T^	0.37	0.15	1.3	—	1.4	—	12	0	0
		*Methylorubrum rhodesianum* D2_2	103741	—	0.03	0.34	—	2.4	—	11	0	0
		*Methylobacterium aquaticum* GR16	16371^T^	0.08	0.02	1.6	—	5.0	—	9	0	0
	*Roseiarcaceae*	*Roseiarcus fermentans* Pf56	24875^T^	—	—	—	—	2.4	—	6	0	0
	*Beijerinckiaceae*	*Methyloferula stellata* AR4	22108^T^	—	—	3.8	—	7.1	—	15	0	0
		*Methylocella palustris* K	n.a.	n.d.	n.d.	n.d.	n.d.	n.d.	—	5	0	0
		*Beijerinckia indica* B.102.C	591	—	—	1.8	—	1.5	—	7	0	0
	*Nitrobacteraceae*	*Rhodoplanes elegans* AS130	11907^T^	—	—	0.09	0.69	0.9	—	17	0	0
		*Variibacter gotjawalensis* GJW‐30	29671^T^	0.91	0.06	—	—	1.0	—	5	0	0
		*Afipia broomeae* B91‐007286	7327^T^	0.12	—	0.20	—	8.3	—	16	43	0
		*Rhodopseudomonas parapalustris* Dr.1	130	—	—	1.2	1.1	2.5	—	10	0	0
		*Bradyrhizobium elkanii* USDA61	11554^T^			0.26	0.61	2.1	—	12	0	0

*Note:* The degree of isomerization at C22 of BHTs and its derivatives as determined by quantification of the 22S and 22R bishomohopan‐32‐ol epimers in the Rohmer degradation mixtures using GC‐MRM‐MS. Also reported are the number of individual BHPDs detected with HPLC‐HRMS^n^ and % pentol plus hexol BHPDs and % unsaturated BHPDs based on these analyses.

Abbreviations: — = concentration is below level of detection, *M* = *Micropepsales*, n.a. = not applicable, n.d. = not determined.

**TABLE 4:** The degree of methylation (%) for C_30_ hopanoids, tetrahymanol, and the Rohmer degradation products, homohopan‐31‐ol and bishomohopan‐32‐ol, for methylation in ring‐A at C‐2 and C‐3 in 52 species of APB^a^ as determined by GC‐MRM‐MS.

Order	Family	Species–strain number	DSM culture	Degree of methylation (%)							
				2‐Methyl						3‐Methyl	
				Hop‐22 (29)‐ene	Hop‐17 (21)‐ene	Diplop‐terol	Tetra‐hymanol	C_31_ hop‐anol	C_32_ hop‐anol	C_31_ hop‐anol	C_32_ hop‐anol
*Rhodospirillales*	*Acetobacteraceae*	*Rhodopila globiformis* 7950	161^T^	—	—	—	—	—	—	—	0.13
		*Komagataeibacter europaeus* JK2	13110	—	—	—	—	—	—	25	4.1
		*Kozakia baliensis* YO‐3	14400^T^	—	—	—	—	—	—	—	0.10
		*Acetobacter pasteurianus* 190	3509^T^	—	—	—	—	—	—	—	0.12
	*Rhodovibrionaceae*	*Tistlia consotensis* USBA 355	21585^T^	—	—	—	—	—	—	—	0.02
	*Rhodospirillaceae*	*Hypericibacter terrae* R5913	109816^T^	—	—	—	—	—	—	—	0.23
*Hyphomicrobiales*	*Parvibaculaceae*	*Rhodoligotrophos appendicifer* 120‐1	23582^T^	29	36	39	—	—	—	—	—
	*Methylobacteriaceae*	*Enterovirga rhinocerotis* YIM 100770	25903^T^	—	—	2.0	—	—	—	—	—
		*Methylobacterium soli*	21955^T^	7.2	53	76	—	—	—	—	—
		*Methylobacterium oxalidis* 35a	24028^T^	14	73	72	—	—	0.10	—	0.19
		*Methylorubrum rhodesianum* D2_2	103741	89	93	91	—	—	0.69	—	—
		*Methylobacterium aquaticum* GR16	16371^T^	17	55	73	—	—	0.66	—	—
	*Beijerinckiaceae*	*Methyloferula stellata* AR4	22108^T^	—	—	23	—	—	0.09	—	—
		*Beijerinckia indica* B.102.C	591	—	—	23	—	—	0.17	—	—
	*Nitrobacteraceae*	*Rhodoplanes elegans* AS130	11907^T^	—	—	18	77	—	—	—	—
		*Afipia broomeae* B91‐007286	7327^T^	—	—	—	—	—	0.06	—	—
		*Rhodopseudomonas parapalustris* Dr.1	130	26	—	34	13	0.93	1.4	—	—
		*Bradyrhizobium elkanii* USDA61	11554^T^	—	—	78	76	3.3	5.1	—	—

^a^ See for all investigated species Table 2; in this Table only the ones where methylation was positively identified using GC‐MRM‐MS are reported. Hopanoid compositions were not analyzed with the Rohmer degradation method for *Komagataeibacter xylinus* R‐2277 and *Methylocella palustris* K. HPLC‐MS^n^ of intact BHPDs revealed that *K. xylinus* contains substantial amounts of 3‐methyl BHPDs and that *M. palustris* shows no signs of methylation of ring‐A of its BHPDs.

Whole cells of 52 of the 54 investigated species (Table 3) were exposed to Rohmer degradation, and the oxidation products of the BHPDs were quantified. This procedure has the advantage that it also enables the detection of hard‐to‐extract BHPDs, but has the disadvantage that it only gives products when two adjacent vicinal hydroxy groups are present in the BHPD, which excludes, for example, adenosylhopanes. All but 9 investigated species gave Rohmer degradation products, with yields ranging over > 2 orders of magnitude (0.1–28 mg g^−1^ biomass; Figure 3c; Table 3). These concentrations are in line with concentrations reported earlier for APB (Rohmer et al. 1984). The 9 species, which apparently do not contain BHPDs because no Rohmer degradation products were detected, included the 5 species that lacked the genetic capability of cyclizing squalene (see above) and *Ensifer sojae* , which contains *shc* but lacks all other genes required for BHPD production (Figure 2b). The other three species that generated no detectable Rohmer degradation products, i.e., *Microvirga massiliensis*, *Azospirillum brasilense* , *Skermanella aerolata* , do possess the genetic capability to produce BHPDs (Figure 2b). BHT derivatives are the major BHPDs in the order *Hyphomicrobiales*, as reflected by an average relative abundance of bishomohopan‐32‐ol of > 97% (Table 3; Figure 3c). In 6 species forming a phylogenetic clade within the family *Acetobacteraceae* of the order *Rhodospirillales*, the 22S epimer of bishomohopan‐32‐ol occurred in high relative abundances (7%–79%; Table 3). In good agreement, BHPDs with a 22S stereochemistry have been reported for BHT and adenosylhopane in *Acetobacter*, *Rhodoblastus*, and *Rhodopseudomonas* spp. (Rohmer and Ourisson 1976a, 1976b; Rohmer et al. 1984; Neunlist et al. 1988). In the orders other than the *Hyphomicrobiales*, bacteriohopanepentol and ‐hexol derivatives (as revealed by the detection of homohopan‐31‐ol and hopan‐30‐ol, respectively) are far more common, with a few exceptions (*Hypericibacter terrae*, *Magnetospirillum fulvum*, *Rhodospirillum rubrum* , *Novosphingobium rosa* , *Sphingomonas changbaiensis* ) (Table 3, Figure 3c). Bacteriohopanehexol derivatives are relatively rare and only occur in the order *Rhodospirillales*, with substantial relative abundances (25%–61%) for *Asaia siamensis* and *Nitrospirillum amazonense*. Mono‐, and in one case, diunsaturated Rohmer degradation products were also detected sometimes in high relative abundance (up to 81% in *Acetobacter pasteurianus* ) (Table 3, Figure 3c). Their occurrence was limited to six species in the order *Rhodospirillales* and one species in the order *Hyphomicrobiales* (Table 3).

**FIGURE 4:**
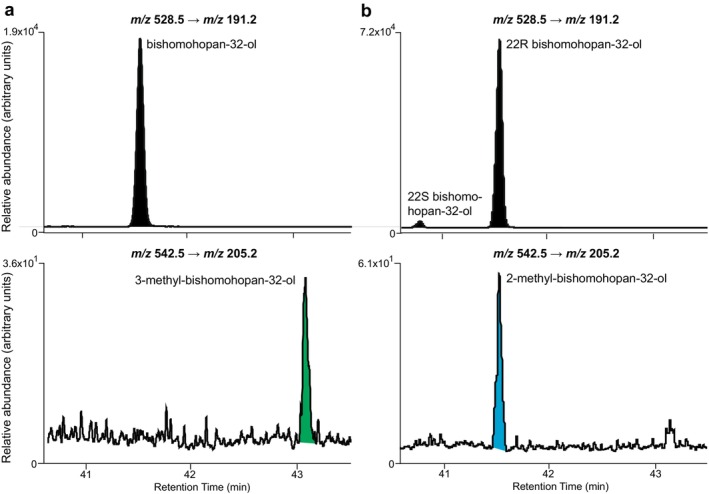
Partial MRM chromatograms revealing how the low abundance of methylated bishomohopanols formed upon Rohmer degradation of cell material of APB was quantified. The transitions *m/z* 528.5 → 191.2 and *m/z* 542.5 → 205.2 shown for (a) *Rhodopila globiformis* 7950 and (b) *Afipia broomea* Dr.1 reveal the presence of bishomohopan‐32‐ol (both species) and 3‐methyl and 2‐methyl bishomohopan‐32‐ol, respectively. Note the relatively low but still quantifiable abundance of the methylated counterparts. Tetrahymanol, diplopterol, and other hopanols were analyzed as their TMS derivatives and the transitions measured are those of the *m/z* M^+•^‐90 fragments to fragments composed of the AB‐ring system of the hopanoid that typically form the major fragment ions. The degree of methylation of the BHPDs in APB species that contain (traces of) methylated BHPDs are reported in Table 4.

In sharp contrast to the C_30_ hopanoids and tetrahymanol, the degree of methylation of the Rohmer degradation products of the BHPDs was much lower (Table 4). To accurately assess the degree of methylation of the hopanols formed upon the Rohmer degradation, they were analyzed with GC–MS triple quad using multiple reaction monitoring (MRM) for a number of specific transitions (Table S1). This allowed for the assessment of the percent of methylation at a detection threshold of ca. 0.01% (see Figure 4 for two examples). The detected 3‐methyl derivatives were dominated by 3‐methylbishomohopan‐32‐ol, which occurs in 7 of the 52 investigated species, mostly in small amounts (% 3‐Me = 0.02–0.27; Table 4) except for *Komagataeibacter europaeus*, where it is more abundant (% 3‐Me = 4.1; Table 4). The only other 3‐methyl derivative detected was 3‐methylhomohopan‐31‐ol, which also occurred in *K. europaeus* in high abundance. The 2‐methyl hopanols partly coelute with the regular hopanols, which makes their quantification with MRM essential. They were detected in 8 species all belonging to the *Hyphomicrobiales* (Figure 2c) with % 2‐Me values ranging from 0.06 to 5.1, with an average of 1.0 (Table 4). Compared with the % 2‐Me values of diplopterol (46%), hop‐22(29)‐ene (30%), hop‐17(21)‐ene (62%), and tetrahymanol (55%), this is a factor 30–60 lower. Hence, C_30_ hopanoids and tetrahymanol seem to be preferentially methylated at C‐2 over BHPDs in the APB species studied. This is in line with the results of Elling et al. (2020), who cultivated the APB *Nitrobacter vulgaris* at a wide variety of cultivation conditions but in all cases also noted a tenfold higher % 2‐Me value for diplopterol than for BHPDs.

### 3.5 BHPD Distributions in APB as Determined by UHPLC‐HRMS^n^ Analyses

In all but 6 (out of 54) of the selected APB cultures, the presence of intact BHPDs was confirmed by UHPLC‐HRMS^n^ (Figures 2c and 3, Table S6). These species were comprised of the 6 species that lacked the genetic capability of producing BHPDs, revealed by the absence of *hpnH* (Figure 2b). Upon Rohmer degradation, 3 additional species generated no detectable BHPD oxidation products, that is, *Microvirga massiliensis*, *Azospirillum brasilense* , *Skermanella aerolata* , but BHPDs were detected by UHPLC‐HRMS^n^ (Figure 2c). For *Azospirillum brasilense* , this is readily explained because the only BHPD detected is adenosylhopane (which does not yield Rohmer degradation products) (Table S6). For the two other species, the reason for this remains unclear but may have to do with the difference in detection limit between the two analytical approaches.

**FIGURE 5:**
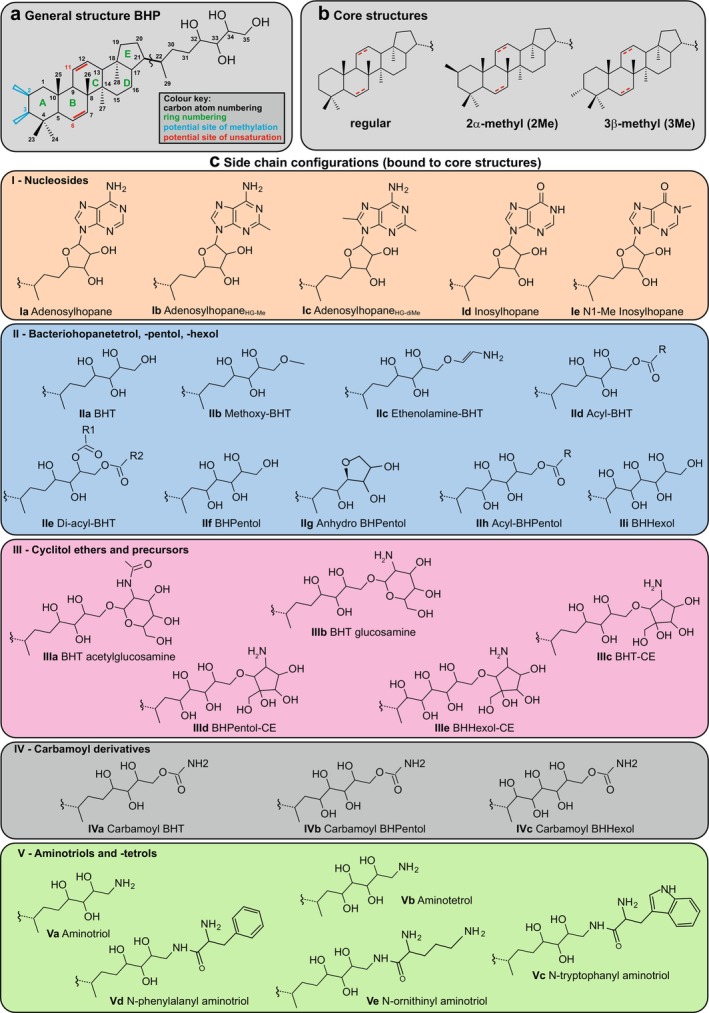
Structures of the identified BHPDs and their applied numbering. (a) General structure of BHT with the indicated sites for structural modification by methylation and unsaturation. The numbering of the carbon atoms and rings is indicated. (b) The three main core structures of the BHPD, that is, without or with methylation in ring A. (c) The variety in the side chains of the BHPDs ordered in five different groups. These can be combined with the cores presented in panel b. The roman numerals can be followed with the extensions “2Me,” “3Me,” or “uns” to indicate ring A methylation or unsaturation.

A wide variety of BHPDs were encountered in the BHPD‐producing APB cultures (see for structures Figure 5). In total, 63 different BHPDs were quantified, and their distribution (Figure 3b, Table S6) will be described according to five different BHPD types: (i) nucleoside BHPDs, (ii) BHT, BHpentol, and BHhexol, and derivatives thereof, (iii) cyclitol ethers (CEs) and precursors, (iv) carbamoyl BHPDs, and (v) aminotriols and ‐tetrols. Ring‐A methylated BHPDs will be discussed as an additional separate group. In Figure 3b they are included in the five different groups of BHPDs, but because of their low abundance (see below), they are hardly visible.

#### 3.5.1 Nucleoside BHPDs

Adenosylhopane (Ia; for structures see Figure 5) is the first intermediate in the biosynthesis of the side chain of BHPDs (Figure 1a; Bradley et al. 2010; Sato et al. 2020, 2021). However, its 22R epimer has also been reported as one of the major BHPDs in the APB *Rhodoblastus acidophila* (Neunlist et al. 1988); small amounts of the 22S epimer were also detected. Our analyses revealed nucleoside BHPDs to be quite common in APB; they occur in 30 out of 48 BHPD‐producing strains in relative abundances of 0.1%–100% (Figure 3b, Table S6). 22R‐adenosylhopane occurs most commonly and abundantly. It is present in 12 of the 21 BHPD‐producing strains belonging to the *Hyphomicrobiales* in relative abundance of 0.9%–89% of total BHPDs and in 3 out of 7 strains of the BHPD‐producing strains belonging to the *Sphingomonadales* in relative abundance of 0.9%–89% (Table S6). In 4 out of 16 BHPD‐producing strains of the *Rhodospirillales*, adenosylhopane was detected in low (< 0.5%) relative abundance. In *Gluconacetobacter diazotrophicus* , *Kozakia baliensis* , *Asaia siamensis* , and *Afipia broomea*, small amounts of the earlier eluting 22S epimer were also detected. This latter species also contained small amounts of presumably Δ^6^ 22R‐adenosylhopane.

Recently, Hopmans et al. (2021) tentatively identified a series of adenosylhopanes that were methylated at the nucleotide moiety in a soil nearby an active methane seep. Similar monomethyl (Ib; 3 isomers) and dimethyl (Ic; 1 isomer) derivatives were detected in *Skermanella aerolata* and, in high abundance, *Rhodoplanes elegans* (Figure 3b, Table S6). Several different *Rhodoplanes* species, including *R. elegans* , have been analyzed before (Lodha et al. 2015), but (methylated) adenosylhopanes were not reported.

Another form of nucleoside BHPs was recently described in a soil nearby a methane seep (Hopmans et al. 2021). These are inosylhopanes where the adenine group is replaced by an inosine moiety. Inosylhopane (Id) was detected in relatively low relative abundances (0.2%–2.7% of total BHPDs) in *Bradyrhizobium elkanii* and *Rhodoplanes elegans* (Table S6). Higher amounts (15.4%) of the N‐methyl inosylhopane (Ie) were found in *Rhodomicrobium vannielii* . Although some of these BHPDs have earlier been detected in the environment (Talbot and Farrimond 2007; Rethemeyer et al. 2010; Kusch et al. 2021; Hopmans et al. 2021; Richter et al. 2023), this is the first comprehensive report of their occurrence in bacterial cultures.

#### 3.5.2 BHT, BHpentol, and BHhexol, and Derivatives

BHT (IIa) and derivatives were detected in 27 of the 46 BHPD‐producing APB in relative abundances of 2%–100% (Figure 3b, Table S6). Two main peaks were detected; the most common one is comprised of the 22R,34R and 22R,34S isomers (see Schwartz‐Narbonne et al. 2020 for detailed discussion), which were not sufficiently separated to be quantified individually. A much earlier eluting (ca. 1.5 min) BHT was identified as the 22S,34S isomer, which has previously been identified in *Komagataeibacter xylinus* (Rohmer and Ourisson 1976b) and *Rhodopseudomonas palustris* , *R. rubrum* and *Rhodoblastus acidophila* (Rohmer et al. 1984). Interestingly, in our studied group of APB species, the occurrence of the 22S,34S isomer of BHT is restricted to the *Rhodospirillales* (Table S4) and occurs in higher relative abundances than previously reported (Rohmer and Ourisson 1976b; Rohmer et al. 1984). The presence of the 22S isomer is confirmed by the detection of 22S bishomohopan‐32‐ol as a Rohmer degradation product in the 6 species within the *Rhodospirillales* that contain this BHT isomer in higher relative abundance (Table 3).

Unsaturated BHTs (Ia‐uns) were encountered with a double bond at Δ^6^ or, in smaller amounts, Δ^11^, and two double bonds (Δ^6,11^). In all cases, the peak consisted of two partially separated BHTs, likely representing the 22R,34R and 22R,34S isomers (see Hopmans et al. 2021), as in the case of the saturated BHT. Peiseler and Rohmer (1992) already identified Δ^6^ monounsaturated BHT in the *Komagataeibacter xylinus* in relatively high amounts (56% of total BHPDs). In our set of APB, the Δ^6^ BHT occurs in relative abundances of up to 53% of total BHPDs (Table S6). The occurrence of Δ^6^ BHT is restricted to species of the *Rhodospirillales* with one exception; in *Afipia broomeae* , which belongs to the *Hyphomicrobiales*, it occurs in a relative abundance of 20%. The Δ^6,11^‐diunsaturated BHT occurs less frequently and less abundantly; it was detected in five species of the *Rhodospirillales* with a relative abundance of 0.5%–9% (Figure 3b, Table S4).

*Enhydrobacter aerosaccus* was the only APB that contained substantial amounts of BHpentol (IIf) and BHhexol (IIi) (16% and 10%, respectively; Figure 3b, Table S6). For all other species, BHpentol was only detected in relatively low amounts (< 0.3%) in a few species. *Hypericibacter terrae* contained a monounsaturated anhydro BHpentol (IIg‐uns) in substantially higher amounts (4%).

Methyl ether derivatives of BHT were tentatively identified in two APB species: *Asaia siamensis* and *Kozakia baliensis* (both belonging to *Rhodospirillales*) contained an unsaturated BHT that was methylated in the side chain (IIb‐Me‐uns) in relative abundances of 8%–30% (Table S6). Kogan et al. (1992) have previously identified a Δ^6^ BHT with a methoxy group at C‐35 in an *Acetobacter* sp., and we assume this is the same BHT. *A. siamensis* also contained its saturated counterpart (IIb) in a relative abundance of 4% (Table S6).

A 35‐ethenolamine BHT (IIc) was tentatively identified in *Methylocella palustris* as the second most abundant BHPD (23%) and in trace amounts (< 1%) in *Beijerinckia indica* (Table S6). To the best of our knowledge, this is the first time that this BHT is identified in a pure bacterial culture. It has previously been reported in soils near a methane seep (Hopmans et al. 2021) and, in trace amounts, in a *Methylobacter*‐*Methylotenera* enrichment and in lake sediments (Richter et al. 2023).

The BHPD distribution of *Hypericibacter terrae* was unusually dominated by BHPDs where one (IId) or two (IIe) of the hydroxy groups in the side‐chain are esterified with fatty acids. Monoacyl BHTs formed the most abundant BHPDs (80%; Table 3b, Table S6).

#### 3.5.3 BHT CE and Intermediates

BHT cyclitol ether (CE; IIIc) is the end product of a three‐step reaction starting with BHT, in which BHT N‐acetylglucosamine (IIIa) and BHT glucosamine (IIIb) are intermediates (Figure 1a). The first identification of BHT CE was in the methylotrophic APB *Methylobacterium organophilum* (Renoux and Rohmer 1985). BHT CE was detected in several species, often in a high relative abundance; in *Novosphingobium rosa* and *Rhizomicrobium palustre* it was the only BHPD detected (Figure 3b, Table S6). Two additional isomers were identified. An earlier eluting isomer, likely the 22S isomer, was only present in *Nitrospirillum amazonense* in a relatively high abundance (45%). A later eluting isomer of unknown structure was present in *Roseiarcus fermentans*, *Rhodoplanes elegans* , *Nitrospirillum amazonense*, and *Novosphingobium nitrogenifigens* in relative abundances of up to 27% (Figure 3b, Table S6). The monounsaturated BHT CE (IIIc‐uns) occurred in two species at trace levels (< 1%; Table S6). A BHT CE containing a methyl group in the CE moiety at an unknown position (IIIc‐Me), previously detected in a lake sediment (Richter et al. 2023), uniquely occurred in *Tistlia consotensis* as the major BHPD (82%) (Figure 3b, Table S6).

BHT N‐acetylglucosamine (IIIa) was detected in 11 of the 48 BHPD‐producing APB in relatively low abundances (1%–11%; Figure 3; Table S6). BHT glucosamine (IIIb) was also a common BHPD; it occurred in 18 of the BHPD‐producing APB. A later eluting isomer of unknown structure (cf. the case with BHT CE) was present in 5 species (Table S6), sometimes in high relative amounts (up to 59%). Monounsaturated BHT glucosamine (IIIb‐uns) occurred only in 2 species at trace levels (< 0.5%). Interestingly, both BHT glucosamine isomers and BHT N‐acetylglucosamine were only detected in species that did not contain BHT CE.

The BHpentol CE (IIId) was detected in 10 species of the *Rhodospirillales* and in 6 species of the *Sphingomonadales*, but not in any species of the *Hyphobacteriales* (Figure 3b, Table S6). Two isomers of monounsaturated BHpentol CE (IIId‐uns; most likely Δ^6^ and Δ^11^) occurred in relative abundances in the range of 2%–14% in 7 species of the *Rhodospirillales*, but not in any of the other APB (Figure 3b, Table S6). The di‐unsaturated counterpart, probably Δ^6,11^, was detected in trace amounts (< 1%) in 3 of these species. The CE derivative of BHhexol (IIIe) was detected in 3 species of the *Rhodospirillales*, sometimes accompanied by mono‐unsaturated counterparts (Table S6).

#### 3.5.4 Carbamoyl BHPDs

BHPDs substituted at C‐35 with a carbamoyl (NH_2_CO‐) group were only present in *Enhydrobacter aerosaccus* , where they formed 55% of total BHPDs (Figure 3b; Table S6). They occurred as derivatives of BHT (IVa), BHpentol (IVb), and ‐hexol (IVc), with the latter two predominating (Table S6). Carbamoyl BHPDs have previously been identified in *Rhodopseudomonas palustris* and *Rhodoblastus acidophila* (Neunlist et al. 1988), also belonging to the *Rhodospirillales*, but only as derivatives of BHT (IVa).

#### 3.5.5 35‐Aminobacteriohopanetriol and Derivatives

35‐Amino BH‐triol (Va) was detected in 15 APB species, all but three (i.e., *Limimonas halophila*, *Tistlia consotensis* , *Sphingomonas formosensis*) belonging to the *Hyphomicrobiales*, varying in relative abundances of 2%–88% (Figure 3b, Table S6). They have been previously structurally identified in two APBs: *Rhodomicrobium vannielii* (Neunlist et al. 1985) and *Methylosinus trichosporium* (Neunlist and Rohmer 1985b). In 4 of these species, a later eluting isomer, which possessed the same MS^2^ spectrum, occurred, sometimes in high relative abundance (81% in *Enterovirga rhinocerotis*; Figure 3, Table S6). This BHPD was also detected in relatively small amounts in *Acidocella aminolytica* and *Stakelama sediminis*, which did not contain the common 35‐amino BH‐triol isomer (Table S6). The Δ^6^ monounsaturated 35‐aminobacteriohopanetriol (Va‐uns) was detected in 4 species with the highest relative abundance in *Afipia broomeae* (Table S6). Their first recognition was in methanotrophic *Methylocaldum* spp. belonging to the *Gammaproteobacteria* (Cvejic et al. 2000).

In *Rhodomicrobium vannielii* , two BHPDs based on the 35‐aminotriol with an amino acid attached to the NH_2_ group via a peptide bond, that is, N‐tryptophanyl aminotriol (Vc) and N‐ornithinyl 35‐aminotriol (Ve), were detected. These BHPDs have been previously identified in another strain of *R. vannielii* (Neunlist et al. 1985). The most abundant (almost 98%) BHPD of *Variibacter gotjawalensis* was also a 35‐aminotriol attached to an amino acid, that is, N‐phenylalanyl 35‐aminotriol (Vd), tentatively identified through its mass spectrum (Figure S1).

Aminotetrol (Vb) was only detected in one species (i.e., *Enterovirga rhinocerotis*) in trace amounts (< 1%; Table S6).

#### 3.5.6 Ring‐A Methylated BHPDs

A variety of 2‐ and 3‐methyl BHPDs was detected but often only in relatively low abundances (Table S6), in agreement with the low degree of methylation of BHPDs as determined by the Rohmer degradation experiments (Table 4). BHPDs methylated at position 2 were encountered in only 3 species falling in the order *Hyphobacteriales*. *Bradyrhizobium elkanii* contained 2‐Me BHT (IIa‐2Me; 2.8%, Table S6), 2‐Me 35‐aminobacteriohopanetriol (Va‐2Me; 3.3%) and 2‐Me adenosylhopane (Ia‐2Me; 0.6%). *Methylorubrum rhodesianum* contained 2‐Me BHT (IIa‐2Me; 7.3%) and *Rhodopseudomonas parapalustris* possessed 2‐Me 35‐aminobacteriohopanetriol (Va‐2Me; 0.9%) and 2‐Me adenosylhopane (Ia‐2Me; 0.1%).

3‐Methyl BHPDs occurred somewhat more frequently. One species of the *Hyphobacteriales*, that is, *Methylobacterium oxalidis*, contained 3‐Me BHT (IIa‐3Me; 2.3%, Table S6) and 3‐Me BHT glucosamine (IIIb‐3Me; 1.1%). The four other 3‐Me BHPD‐producing APB come from the *Rhodospirillales*. *Hypericibacter terrae* only produces 3‐Me BHT (IIa‐3Me; 1.0%), whereas *Kozakia baliensis* produces the saturated (IIIc‐3Me; 0.5%) and monounsaturated 3‐Me BHpentol CE (IIIc‐3Me‐uns; 1.7%), in addition to minor amounts (0.2%) of 3‐Me BHT (IIa‐3Me). The two *Komagataeibacter* species produce a wider variety of 3‐Me BHPD and in much higher relative abundances; 3‐Me BHT (IIa‐3Me) and its mono‐ and diunsaturated counterparts (IIa‐3Me‐uns) form 4.6% to 10.2% of the total BHPDs, while the mono‐ and diunsaturated 3‐Me derivatives of BHT CE (IIIc‐3Me‐uns) and BHpentol CE (IIId‐3Me‐uns) amount to 3%–6% and 21%–29%, respectively. These two latter species are by far the most prominent ring‐A methylated BHPD producers.

## 4 Discussion

### 4.1 Implications for the Biosynthesis of Hopanoids and BHPDs by APB and Their Use in Chemical Taxonomy

#### 4.1.1 Predictability of Hopanoid Production Based on Genome Composition

Importantly, our study of carefully selected APB species revealed that if a species possesses *shc* in its genome, it is likely to actually produce BHPDs; of the 49 cultivated APB species that possess *shc*, all but one (i.e., *Ensifer sojae* ) produced detectable amounts of BHPDs, and the genome of this latter species was actually lacking *hpnH* and other genes of the *hpn* BGC (Figure 2). This indicates that it is likely safe to couple the possession of the *hpn* BGC to actual BHPD production. This is in good agreement with a study on the presence of *hpn* genes and BHPD production in a large set of *Acidobacteria* that showed an excellent match in pheno‐ and genotype in terms of hopanoid production (Sinninghe Damsté et al. 2017). The match is less evident for C_30_ hopanoid production; in 19 out of the 49 cultivated APB species that possessed *shc* in their genomes, BHPDs were detected, but C_30_ hopanoids were absent. This contrasts with another C_30_ triterpenoid, tetrahymanol; the 3 species that possessed *ths* produced tetrahymanol (Figure 2). This is most likely because tetrahymanol is an end product (Banta et al. 2015), not further used to biosynthesize any other natural product, whereas diploptene is an intermediate in the biosynthesis of BHPDs (Figure 1a).

The demonstrated match in pheno‐ and genotype in terms of hopanoid production for the set of 54 APB studied indicates that this most likely can be extrapolated to the set of ca. 2700 APB genomes that do possess the *shc* gene (Table 1). On the one hand, this supports our earlier suggestion (see above) that ca. one quarter of the APB species produce BHPDs because they possess the genetic capacity to do so. On the other hand, it implies that BHPD biosynthesis is scattered through the phylogenetic tree of APB. It is unevenly distributed over the various orders of APBs, that is, it predominantly occurs in the *Rhodospirillales* and, to a lesser extent, in the *Hyphomicrobiales*, and is much more restricted in the other orders (Table 2). At the genus level, there is also a high variability in the ability to biosynthesize BHPDs since the number of genomes encoding GyrB was often (much) larger than those encoding Shc and HpnH (Table 2), and the genomes of many genera of the *Rhodospirillales* and *Hyphomicrobiales* do not possess the *shc* gene (see footnotes in Table 2). Hence, the ability to biosynthesize BHPDs occurs in a scattered way. This can be illustrated by the GyrB phylogenetic tree of over 400 genomes of *Sphingomonas* spp., where the 77 species with a genome containing *shc* are marked in yellow, revealing the highly scattered occurrence of BHPD biosynthesis in this genus (Figure S2). We confirmed this experimentally; in 1 out of 6 examined *Sphingomonas* spp., the *hpn* BGC was absent in the genome and BHPD was indeed not produced (Figure 2). Clearly, the genes of the *hpn* BGC are part of the pangenome but not of the core genome of the genus *Sphingomonas*. At the same time, the proteins encoded by the *hpn* genes in the *Sphingomonas* spp. are phylogenetically closely related, and the architecture of their *hpn* BGCs is highly comparable. This suggests that the *hpn* BGCs in *Sphingomonas* spp. are most likely inherited from a common ancestor and not obtained through horizontal gene transfer. This also holds in general for the *hpn* genes. Phylogenetic trees of the proteins encoded by these genes reveal, in general, a similar clustering in classes, families, and genera as the 16S rRNA (Hördt et al. 2020; Figure 2a for a tree of the studied species) and GyrB (not shown) trees, with no clear evidence of horizontal gene transfer from within or outside the APB. This would imply a common ancestor that contained an *hpn* BGC. However, during evolution, the *hpn* BGC is apparently easily lost from the genome, resulting in its scattered distribution in the extant population of APB. This indicates that BHPD production is not essential for the survival of the APB cell and the *hpn* BGC is probably only kept when it is beneficial for survival. An extreme case is the genus *Sphingobium*, where only 2 out of the examined 123 genomes contain the *hpn* BGC. One of these species, *S. estronivorans*, was tested in this study for BHPD production and still produced BHPDs (Figure 3), indicating its *hpn* BGC was fully functional. The presence of BHPD in these two *Sphingobium* spp. cannot be readily explained by specific physiological or biochemical characteristics in comparison with other *Sphingobium* species.

#### 4.1.2 BHPD Composition: Phenotype in Relation to Genotype

A wide variety of BHPDs was identified in the APB analyzed; 63 different BHPDs were detected (Figure 3b, Table S6). These included well‐known BHPDs (i.e., BHT) known to occur in APB but also BHPDs that have not previously been identified in APB or other bacteria as mentioned earlier. The marked changes in the BHPD distributions are not always clearly coupled to the taxonomy or specific phylogenetic clusters of the APB (cf. Figure 3a,b). The interpretation of these differences is complicated by the fact that only a limited set of the genes involved in the biosynthesis of BHPDs has been fully characterized (see Figure 1a). For example, it is currently unknown which genes are involved in the production of BHPentol and ‐hexol, even though these BHPDs or derivatives thereof may form a substantial part of the bacterial BHPD inventory (see Kusch and Rush 2022 for a review). With the ever‐increasing analytical abilities to identify BHPDs in complex mixtures (e.g., Talbot et al. 2001, 2007a; Hopmans et al. 2021), additional BHPDs will be identified for which no known biosynthetic pathway (with the complementary set of genes) exists. The present study is a good illustration of this. Potential genes may be predicted by the annotation of the genomes, but this remains tentative at best. In the case of BHPD synthesis, one would suspect that such unknown genes would be in close association with the *hpn* BGC, as most known BHPD genes are organized this way in the genomes of APB (Figure 1b). However, there are exceptions to this: *hpnO*, *hpnP*, and *hpnR* are often not part of the *hpn* BGC. During this study, special attention was focused on this aspect, but no obvious candidate BHPD genes (other than the genes encoding HpnA, HpnB, HpnL, and HpnM, which are suspected to function in the biosynthesis of BHPD) in the vicinity of the *hpn* BGC were evident. For the most commonly occurring BHPDs in APB, we will discuss potential relationships between groups of BHPD and genes (potentially) encoding steps in their biosynthesis.

##### 4.1.2.1 C_30_ Hopanoids

The first step in the biosynthesis of BHPDs is the conversion of the C_30_ hopanoids to adenosyl BHPD catalyzed by HpnH (Bradley et al. 2010). This conversion is not always complete, as has been reported in many previous studies. It varied both between and within orders: 92%–100% (average 96%; *n* = 17) for the *Rhodospirillales* species, 32%–100% (average 87%; *n* = 8) for the *Sphingomonadales* species, and 32%–100% (average 73%; *n* = 17) for the *Hyphomicrobiales* APBs. This indicates that, at least in some species, the C_30_ hopanoids also fulfill another biochemical role than just being a precursor for BHPDs. This is also suggested by their often much higher degree of methylation at C‐2 than the BHPDs (see below).

##### 4.1.2.2 Nucleoside BHPDs

It is noteworthy that many APB produce adenosyl BHPDs in high relative abundance (i.e., 9 out of 54 species, mostly belonging to the *Hyphomicrobiales*, contained > 20%), because it is generally considered to be an intermediate on the way to more common BHPDs (Bradley et al. 2010; Figure 1). All these species still contained the genes encoding the proteins to convert adenosyl BHPDs into other BHPDs. Hence, it is likely that they also fulfill a specific role in tuning the physiological properties of the membrane and that BHPD synthesis can be regulated to be stopped in an early phase. In good agreement, Eickhoff et al. (2013) reported already high relative amounts (61%–97% of all BHPDs) of adenosylhopane in *Rhodopseudomonas palustris* and surmised another specific function than its exclusive role as an intermediate in BHPD biosynthesis.

##### 4.1.2.3 22S Diastereomers

Both the results of the Rohmer degradation and the UHPLC‐MS^n^ analysis revealed the presence of relatively abundant 22S diastereomers in species falling in a phylogenetic clade within the family *Acetobacteraceae*. Small amounts of (22S)‐diastereomers have previously been reported in, for example, acetic acid bacteria (Rohmer et al. 1984) and *Rhodoblastus acidophila* (Neunlist et al. 1988), but the relative abundances are much higher in this study with a 22S/(22S + 22R) ratio as high as 0.79 in *Gluconacetobacter diazotrophicus* (Table 3) and 22S BHT often occurring in higher abundances than the common 22R BHT (Table S6). Sato et al. (2021) showed that the 22R‐configuration in BHPD biosynthesis in *Streptomyces* and *Zymomonas* is determined by the function of HpnH (i.e., the first step in BHPD biosynthesis; Figure 1a), especially by the identity of the amino acid at position 106 of this protein. When the common cysteine at this position was replaced by alanine in expression experiments with *E. coli* , both 22R‐ and 22S‐adenosylhopanes were biosynthesized. However, these authors already noted that HpnH homologs derived from *Rhodoblastus*, *Rhodopseudomonas*, and *Rhodospirillum* spp. have the conserved cysteine residue corresponding to Cys106 in HpnH of *Zymomonas* sp., and the same holds for *Gluconacetobacter* (data not shown). Hence, other, yet unknown, factors determining the HpnH 3D‐structure must be responsible for the reduced stereospecificity in catalyzing the formation of adenosylhopane by HpnH.

##### 4.1.2.4 BHPD CE and Its Precursors

A good match exists in our database concerning the occurrence of the *hpnI*, *hpnJ*, and *hpnK* genes and the production of In 31 out of the 36 strains that produced BHPD CE‐type components, *hpnI*, *hpnJ*, and *hpnK* were positively identified in their genomes (cf. Figure 2b,c). In the genomes of four species (i.e., *Rhodospirillum rubrum* , *Skermanella aerolata* , *Microvirga massiliensis*, and *Afipia broomeeae*), these genes were not detected, which remains unexplained but may relate to the incompleteness of their genomes. *Rhodoligotrophos appendicifer* lacks *hpnJ* in its genome (Figure 2b), and its BHPD distribution is 100% composed of BHT glucosamine (Figure 3, Table S6), which is fully consistent with the fact that HpnJ catalyzes the last step in the production of BHPD CE (Figure 1a). However, many other APB that contain the full assemblage of the three genes still contain BHT glucosamine as a dominant (up to 90%) BHPD. Apparently, the properties of BHT glucosamine and BHT CE are different, and we interpret that this is used by the APB to adjust their relative abundance to tune the physical properties of their membranes. *Methylocella* spp. lacks both *hpnJ* and *hpnK* in its genome but possesses *hpnI* (Figure 2b), although it does not contain BHT acetylglucosamine as would be expected based on the biosynthetic scheme (Figure 1a). However, this is in line with the observation that BHT acetylglucosamine is typically only present in trace amounts (< 5%) or absent, with only 3 species having a slightly higher relative abundance (7%–11%) (Figure 3; Table S6). This suggests that BHT acetylglucosamine is primarily an intermediate and not a preferred membrane rigidifier for APB.

##### 4.1.2.5 Amino BHPDs

The *hpnO* gene encoding the aminotransferase HpnO, which is responsible for the formation of amino BHPDs (Figure 1a), is restricted to the *Hyphomicrobiales*, although not all species possess it (Figure 2b, Table S5). Typically, *hpnO* is not associated with the *hpn* BGCs. It is surprising to note that the production of amino BHPDs occurs more commonly than that of *hpnO* (cf. Figure 2b,c). Production of amino BHPDs also occurs outside the *Hyphomicrobiales* (Figure 2c), with the most prominent example being *Limimonas halophila*, where 35‐aminotriol is the only BHPD detected. A partial explanation may be that the distinction between HpnO and other proteins of the aminotransferase family is small, and proteins annotated as “aspartate aminotransferase family protein” with *E* values > 1*E* ^−100^ may also catalyze this reaction (Table S5).

##### 4.1.2.6 BHpentols and ‐Hexols

BHpentols and ‐hexols and their derivatives only occur in species belonging to the *Rhodospirillales* and *Sphingomonadales* but not in the *Hyphomicrobiales* (Figure 3, Table S6). As mentioned, the genes encoding the enzymes to produce these BHPDs are unknown, hindering the comparison of BHpentol and ‐hexol production with genomic data. Based on the known scheme of BHPD biosynthesis (Figure 1a), one may speculate that their biosynthesis occurs by (repeated) hydroxylation of the CH_2_ group(s) adjacent to the tetrol moiety of BHT. However, hydroxylases that are able to bind a hydroxy group to an sp^2^ carbon atom are, to the best of our knowledge, not known. Alternatively, the biosynthesis could be performed in two (or four) steps: first, an oxidation of BHT resulting in a 32‐oxo group, followed by water addition to its enol form by a hydratase. This is comparable to the mechanism proposed for the formation of 31‐methyl BHPDs, where the last step is a methylation of the Δ^31^ double bond instead of the addition of water (Simonin et al. 1994). In favor of this second possibility is that a potential intermediate, that is, 32‐oxobacteriohopane‐33,34,35‐triol glycoside, has been identified as a minor BHPD in the APB *Zymomonas mobilis* (Flesch and Rohmer 1989). This sequence of reactions could be repeated to produce BHhexols. Considering this together with the knowledge about the capacity to produce BHpentols and ‐tetrols in the APB studied (Figure 3), the vicinity of the *hpn* BGCs were monitored for potential genes encoding proteins potentially enabling these reactions. Unfortunately, no potential candidate genes were identified. Detailed bioinformatic and biochemical studies will be required to solve this issue.

##### 4.1.2.7 Unsaturated BHPDs

The identification of unsaturated BHPDs with double bonds at the 6 and 11 positions (or both) has been reported previously in APB: in two strains of *Methylosinus* spp. (Rohmer et al. 1984), in an *Acetobacter* sp. (Rohmer and Ourisson 1986), in *K. xylinus* (Peiseler and Rohmer 1992), and in *Gluconacetobacter xylinus* (Talbot et al. 2007b). However, the genes encoding proteins involved in desaturating the BHPDs (which seem the most logical biochemical pathway to produce them) are currently unknown. Interestingly, several of the investigated APB contain a gene that is annotated as a “sterol desaturase family protein” encoding a protein composed of ca. 260 amino acids (e.g., WP_003625306.1 of *Acetobacter pasteurianus* ) not belonging to the *hpn* BGCs but located elsewhere on the genome. Since the investigated APB do not produce sterols (as most bacteria), this protein may be responsible for the introduction of one of the double bonds in BHPDs sometimes present in APB. The gene encoding this desaturase occurs in ca. two thirds of the examined *Rhodospiralleles* spp., in half of the examined *Sphingomonadales* spp., but not in the examined *Hyphomicrobiales* spp. (Figure 2b, Table S5). This roughly matches with the occurrence of unsaturated BHPDs in the APB (Figure 2c), but the match is not 1:1. There are APB that do produce unsaturated BHPDs but do not possess the genes encoding the desaturase. For example, *Afipia broomea*, falling in the *Hyphomicrobiales*, contains 20% Δ^6^ BHT and 21% Δ^6^ aminotriol (Figure 3f, Table S6), but the gene encoding this desaturase was not detected. The protein identified here is not the previously identified “sterol desaturase family protein” of *Rhodomicrobium udaipurense* of similar size, which is encoded by a gene designated *hpnX* (Tushar et al. 2014; Table S2). The occurrence of this gene is much more limited and occurs only in *Rhodomicrobium* spp. Hence, more research is required to resolve the question of which genes are responsible for the formation of unsaturated BHPDs.

##### 4.1.2.8 Methylated BHPDs

The link between the presence of the genes encoding the proteins for methylation at C‐2 of the A‐ring of the C_30_ hopanoids, tetrahymanol, and the BHPDs is quite evident. If an APB species produces C_30_ hopanoids, tetrahymanol, or BHPDs methylated at C‐2, then *hpnP* is present in its genome (Figure 2b,c). The opposite is not true: our study identified 2 species ( *Hyphomicrobium facile* , *Methylocella* sp.) containing the *hpnP* gene but lacking the corresponding 2‐methyl product(s). In the case of *Methylocella*, this was not tested with the sensitive MRM method performed on the Rohmer degradation products, so this remains tentative. Although we used a sensitive analytical method to determine the degree of ring‐A methylation of BHPDs in *H. facile* , we cannot fully exclude that this was due to the cultivation conditions used, since it has been demonstrated that culture conditions can influence the degree of methylation of hopanoids (Rashby et al. 2007; Eickhoff et al. 2013; Elling et al. 2020, 2022). Nevertheless, we must conclude that the presence of *hpnP* in the genome does not always result in detectable 2‐methyl hopanoid production.

The *hpnR* gene was detected in 12 of the 54 APB species studied and was less restricted to a specific order than in the case of the *hpnP* gene (Figure 2). Remarkably, 4 out of 12 species in this group (i.e., *Gloconoacetobacter diazotrophicus*, *Gloconobacter oxydans*, *Microvirga massiliensis*, and *Methylobacterium soli*) did not produce 3‐methyl hopanoids (Figure 2b,c), again indicating that the presence of genes encoding enzymes for hopanoid ring‐A methylation does not guarantee that they actually use these genes. In contrast to HpnP, HpnR was only used to methylate BHPDs and not C_30_ hopanoids in the case of the studied species.

Two of the examined species possess both *hpnP* and *hpnR* in their genomes, that is, two of the three *Methylobacterium* strains studied (Figure 2b). Sinninghe Damsté et al. (2017) identified earlier an acidobacterium (*Ca*. Koribacter versatilis) that possessed both ring‐A methylation genes and showed that it produced BHPDs substituted at both C‐2 and C‐3. In the case of the *Methylobacterium* spp., however, such BHPDs were not identified, neither intact nor by using the Rohmer degradation method.

### 4.2 Geobiological Implications

#### 4.2.1 Interpretation of the Sedimentary Hopanoid Record

Pearson et al. (2007) concluded on the basis of an analysis of environmental *shc* sequences that probably < 10% of the bacterial species are capable of hopanoid production, despite the ubiquity of dia‐ and catagenetic products of BHPDs and C_30_ hopanoids in sediments and petroleum (e.g., Ourisson and Albrecht 1992). A follow‐up study (Pearson and Rusch 2009), in which almost 10 million shotgun sequences from oceanic environments were analyzed, reduced this number to < 4%. However, they showed that one third of the retrieved *shc* sequences could be attributed to APB, suggesting that APB may form an important group of BHPD producers in the marine environment (Pearson and Rusch 2009). Our comprehensive study of > 6000 reference genomes of APB revealed that ca. 23% of the APB species possess the genetic capacity to produce BHPDs, which is indeed substantially higher than estimated for all marine bacteria (< 4%; Pearson and Rusch 2009) and would support the idea that APB form an important group of BHPD producers in the environment. However, it should be realized that the species in this study mainly derive from the terrestrial environment (Table 1), and it remains unsettled if our findings can directly be related to marine environments.

Two other general, less positive points for the interpretation of the fossil BHPD record arise from our study. First, the BHPD concentrations in APB species that do produce hopanoids can vary by two orders of magnitude (Figure 2c, Table 3), and BHPD concentrations are not directly related to a specific phylogenetic group, even though we acknowledge that these data are limited by the use of the Rohmer degradation method, which does not quantify nucleoside BHPs, and may be affected by growth conditions and growth stage. Nevertheless, the overall consequence of these findings is that in the environment the origin of the BHPDs may be biased towards specific species that produce relatively high amounts of BHPDs. It would therefore, for example, still be unsafe to predict which bacteria are important BHPD producers based on the presence of the *shc* or *hpnH* in environmental metagenomes (cf. Pearson and Rusch 2009). Second, the BHPD BGC is not evenly distributed over taxonomic/phylogenetic groups as one would expect when this trait was only primarily obtained by vertical gene transfer, and BHPD synthesis would be a conservative trait. In that case, one would expect to see that specific phylogenetic clades would possess the BHPD BGC, while others lack them. This is confirmed by the basic congruence of the phylogenic trees of the various *hpn* genes of the *hpn* BGCs in comparison with that of a conservative gene such as *gyrB*. This would suggest that the *hpn* BGC was already present in the common ancestor of the APB. However, only ca. 23% of the APB still contains an *hpn* BGC (Table 2), indicating a relatively easy loss of the *hpn* gene cluster during evolution. This phenomenon is also observed at the genus level (see the examples described above of *Sphingomonas* and *Novosphingobium*). This means that we cannot predict the production of BHPDs in one species by analyzing a closely related species. It will be even harder to predict on the basis of the presence or absence of BHPDs in extant microbes if related species in the geological past possessed these or not. This complicates the use of BHPDs as biomarkers in environmental and paleontological studies.

#### 4.2.2 2‐Methyl Hopanoids as Biological Markers

2‐Methyl hopanoids have been used in evolutionary studies as markers for the advent of oxygen‐producing cyanobacteria (Summons et al. 1999). However, their production by an APB, that is, the anoxygenic phototroph *Rhodopseudomonas palustris* , prompted Rashby et al. (2007) to warn that the potential origins of sedimentary 2‐methyl hopanoids are more diverse than previously thought, although in the last three decades of the previous century the presence of these types of hopanoids was already demonstrated in many other bacteria (see Rohmer 1993 for a review). Nevertheless, this study further stimulated research on this topic, and the presence of the *hpnP* gene in the genomes of bacteria and in the environment, in some studies combined with confirmed production of 2‐methyl C_30_ hopanoids and BHPDs, was demonstrated (e.g., Welander et al. 2010; Ricci et al. 2014, 2015; Sinninghe Damsté et al. 2017; Elling et al. 2020, 2022), casting doubt on the interpretation of 2‐methyl hopanoids as biomarkers for cyanobacteria. Hoshino et al. (2023) recently showed that *hpnP* was present in the last common ancestor of the cyanobacteria, while this gene appeared in APB only around 750 Ma ago. Hence, these authors concluded that in sediments older than 750 Ma, it is probably still safe to use 2‐methyl hopanoids as a biomarker for cyanobacteria. Identification of environmental *hpnP* sequences further indicated that in specific environments, cyanobacteria are the major source for this gene sequence and presumably for 2‐methyl hopanoids produced in these environments (Ricci et al. 2014, 2015; Garby et al. 2022).

Apart from Proterozoic (> 550 Ma) sediments (Summons et al. 1999), high relative abundances of 2‐methyl hopanoids have also been reported for marine sediments deposited during Cretaceous oceanic anoxic events (OAEs) (Kuypers et al. 2004). During these events, vast amounts of organic matter were buried in so‐called “black shales”, which have served as petroleum source rocks for a substantial part of the fossil fuel extracted and utilized to date. Oceanic anoxia created by special conditions played an important role in the preservation of organic matter in these black shales. For example, the Cenomanian proto‐North Atlantic, the dominant spot of OAE‐2 black shale deposition, was a huge Black Sea‐type of basin with restricted connection to other ocean basins and euxinic conditions reaching into the photic zone (e.g., Sinninghe Damsté and Köster 1998; Kuypers et al. 2002). Remarkably, the % 2‐methyl values of extended (i.e., C_30+_) hopanoids in OAE black shales were unusually high and sometimes reached over 30% (Kuypers et al. 2004). At the same time, these sediments showed depleted ^15^N/^14^N ratios, indicative of cyanobacterial dinitrogen fixation. This complementary evidence was used to infer that pelagic cyanobacteria were the predominant source for 2‐methyl hopanoids in OAE black shales (Kuypers et al. 2004) following Summons et al. (1999). This hypothesis was supported by the correlation of the abundance of 2‐methyl anhydroBHT with ^15^N/^14^N ratios in OAE‐2 black shales from Germany (Blumenberg and Wiese 2012). The nitrogen isotopic composition of fossil porphyrins in OAE‐2 black shales provided further support that N_2_‐fixing cyanobacteria were major primary producers during that time (Ohkouchi et al. 2006). An alternative hypothesis for the high relative abundances of 2‐methyl hopanoids in OAE‐2 black shales was proposed by Elling et al. (2020). They showed by extensive cultivation experiments that the nitrite‐oxidizing APB *Nitrobacter vulgaris* produced high but variable relative amounts of 2‐methyl hopanoids and hypothesized that related marine nitrifiers played an important role in nitrogen cycling at the redoxcline in the stratified oceans during the OAEs and hence were the predominant producers of 2‐methyl hopanoids. The choice between these two alternative explanations is complicated by the fact that neither 2‐methyl BHPD‐producing cyanobacteria nor *Nitrobacter* spp. have been identified in present‐day open marine environments.

What does this study contribute to our understanding of the cause of the high relative abundances of 2‐methyl hopanoids on OAE black shales? Our compiled data confirm that the occurrence of 2‐methyl hopanoid production in APB is almost completely restricted to seven families (i.e., *Beijerinckiaceae*, *Nitrobacteraceae*, *Hyphomicrobiaceae*, *Lichenihabitantaceae*, *Methylobacteriaceae*, *Methylocystaceae*, and *Parvibaculaceaea*; Tables 2 and 3) of the order *Hyphomicrobiales* (cf. Elling et al. 2020; Hoshino et al. 2023). Elling et al. (2020) proposed that the 2‐methyl hopanoids during the deposition of OAE black shales would only be produced by marine relatives of the nitrifier *Nitrobacter vulgaris* . However, the group of 2‐methyl hopanoid‐producing *Hyphomicrobiales* is comprised of species with a diverse physiology (i.e., not only nitrifiers but also methylotrophs, anoxygenic phototrophs, heterotrophs, etc.), so the presence of 2‐methyl hopanoids in the fossil record cannot be interpreted with any certainty to reflect a predominant origin from a bacterial species with a specific physiology. Furthermore, with the interpretation of the hopanoid sedimentary record, a “dilution effect” should always be considered. BHPDs are estimated to be produced by ca. 4%–10% of the marine bacterial community (Pearson et al. 2009; Pearson and Rusch 2009). Hence, in the environment, production of 2‐methyl hopanoids by a group of highly specific bacterial species (e.g., nitrifying APB) will always be accommodated by a presumably much larger production of a wide group of bacterial species producing only non‐methylated BHPDs. In the sedimentary record, this must result in substantially reduced relative abundances of 2‐methyl hopanoids and, hence, a much lower 2‐methyl index of extended hopanoids. In addition, an important observation from this study is that in the studied *Hyphomicrobiales* species, the degree of C_2_ methylation is always much higher for C_30_ hopanoids than for the BHPDs: for three APB species that produced 2‐methyldiplopterol, no 2‐methyl BHPDs were detected at all (Table 4), whereas for the 7 species that did contain 2‐methyl BHPDs, the degree of C_2_ methylation of the BHPDs was 1–3 orders of magnitude lower than that of diplopterol (Table 4). This much reduced degree of 2‐methylation of BHPDs compared with C_30_ hopanoids is consistent with earlier findings in APB (e.g., Vilchèze et al. 1994; Rashby et al. 2007; Eickhoff et al. 2013; Elling et al. 2020). The one exception to this general observation is *Afipia broomeae* , where diplopterol did not occur as a 2‐methyl derivative, but where the 2‐methyl BHT was detected, albeit in extremely low abundance (0.06%, see Table 4). The geobiological implications of the observed much lower degree of C‐2 methylation of BHPDs in comparison with C_30_ hopanoids have not been fully realized since no discrimination is made between reported sedimentary 2‐methyl hopanoid indices for C_30_ hopanes (e.g., Summons et al. 1999; Hoshino et al. 2023), for extended hopanoids (e.g., Kuypers et al. 2004; Blumenberg and Wiese 2012), or a mix of both (Naafs et al. 2022). Hopanes are formed from BHPDs by dia‐ and catagenetic reactions in sediments and rocks. These reactions often result in a breakdown of the extended side‐chain of BHPDs (e.g., see Sinninghe Damsté et al. 1995). The consequence is that BHPDs may act as (additional) precursors for C_30_ hopanes, but the opposite is not true: BHPDs are the only precursors for extended hopanes. Hence, the high % 2‐methyl values of extended hopanoids (sometimes > 30%) reported for the OAE black shales (Kuypers et al. 2004; Blumenberg and Wiese 2012) should be compared with those of BHPDs—and not with those of C_30_ hopanoids—of APB cultures, which range from 0% to 5.1% (average value 0.7%) for the 12 out of 54 species of APB in our study set that do produce 2‐methyl hopanoids (Table 4). For *Nitrobacter vulgaris* , they range from 0.2% to 5.6% (average value ca. 2%), depending on growth conditions (Elling et al. 2020).

The bottom line of these three considerations is that the production of 2‐methyl BHPDs by APB cannot explain the reported high 2‐methyl hopanoid indices of extended hopanoids in OAE black shale (Kuypers et al. 2004; Blumenberg and Wiese 2012). In contrast to the APB, the degree of methylation at C‐2 of BHPDs of cultures of cyanobacteria as determined by Rohmer degradation can be much higher (reaching values of up to 100%) (Summons et al. 1999 and references cited therein). Therefore, we deem the hypothesis of Elling et al. (2020) that marine nitrite‐oxidizing bacteria closely related to *N. vulgaris* are the predominant source for 2‐methyl hopanoids in OAE black shales unlikely. We agree that there are also large issues with the idea that pelagic N_2_‐fixing cyanobacteria explain the very high 2‐methyl index of extended hopanoids in OAE black shales because there is no contemporary evidence for 2‐methyl hopanoid‐producing N_2_‐fixing cyanobacteria in open marine environments. However, this hypothesis can at least explain the depleted ^15^N/^14^N ratios characteristic for these black shales, which is an independent marker of cyanobacterial dinitrogen fixation. Furthermore, the production of 2‐methyl hopanoids by a dominant fraction of the phytoplankton (i.e., photosynthetic cyanobacteria), rather than a small bacterial population of nitrite‐oxidizing bacteria at the chemocline, is a more convincing explanation since sedimentary marine organic matter is primarily derived from phytoplanktonic remains and hence the “dilution effect” plays a less important role.

#### 4.2.3 3‐Methyl Hopanoids as Biological Markers

For a long time, 3‐methyl hopanoids have been used as biomarkers for aerobic methane oxidation because they occur abundantly in so‐called Type I methanotrophic bacteria (see Kusch and Rush 2022 for a review). However, Welander and Summons (2012) showed that not all methanotrophs possess *hpnR* and that this gene is also found in other bacterial species. Production of 3‐methyl BHPDs was already demonstrated much earlier in acetobacteria (e.g., Zundel and Rohmer 1985) and later on in phototrophic purple nonsulfur bacteria (Mayer et al. 2021).

Our work shows the occurrence of 3‐methyl BHPDs in seven species of the *Rhodospirillales* (Figure 2). In contrast to 2‐methyl hopanoids, no 3‐methyl C_30_ hopanoids were detected. Relative abundances of 3‐methyl BHPDs are low (degree of methylation < 0.25%; Table 4), except for *Komagataeibacter* spp. None of these species are aerobic methanotrophs: the *Komagataeibacter* spp., *Kozakia baliensis* , and *Acetobacter pasteurianus* ferment acetic acid; *Tistlia consotensis* is an aerobic, chemoheterotrophic, nitrogen‐fixing APB, *Hypericibacter terrae* is an aerobic/microaerophilic heterotroph, and *Rhodopila globiformis* is a phototrophic purple nonsulfur APB. All these species possessed the *hpnR* gene, but there were two more species within the *Rhodospiralles* that possessed *hpnR* in their genomes that did not produce 3‐methyl BHPDs under the culture conditions employed (cf. Figure 2b,c). This confirms our conclusion with respect to *hpnP*: the presence of a gene encoding a protein catalyzing methylation of the A‐ring of a hopanoid cannot be taken as solid proof of actual production of methylated hopanoids. Hence, one cannot be sure about the actual production of methylated hopanoids only on the basis of genome analysis. Within the *Hyphomicrobiales*, there were three species that possessed *hpnP*, but only one of them (*Methylobacterium oxalidis*) produced 3‐methyl BHPDs. Surprisingly, the *Methylobacterium* spp. also possessed *hpnP* and produced 2‐methyl BHPDs. However, no evidence was found for the production of 2,3‐dimethyl BHPDs, as has been reported for an acidobacterium possessing both *hpnP* and *hpnR* (Sinninghe Damsté et al. 2017). Overall, our study confirms the production of 3‐methyl BHPDs by APB other than those oxidizing methane aerobically, supporting the use of additional methods (e.g., their stable carbon isotope composition; for example, Jahnke et al. 1999; Cordova‐Gonzalez et al. 2020) to examine when sedimentary 3‐methyl hopanoids are linked to the past occurrence of aerobic methanotrophy.

## 5 Conclusions

(1) Genome analysis of > 6000 reference genomes of APB revealed that ca. 23% possess the genetic capacity to produce BHPDs, substantially higher than for all bacteria, supporting the idea that the APB form an important group of BHPD producers in the environment.

(2) However, BHPD biosynthesis genes were unevenly distributed between taxonomic and phylogenetic groups and not consistently found in monophylogenetic clusters. This undermines the use of the presence/absence of gene encoding proteins catalyzing specific biosynthetic pathways to infer biological sources of BHPDs and their dia‐ and catagenetic products in a palaeobiological and palaeoenvironmental context.

(3) Analysis by UHPLC‐MS^n^ of intact BHPDs in 54 cultivated strains of the three major orders of the APB (*Hyphomicrobiales*, *Rhodospirillales*, and *Sphingomonadales*), including species of 29 genera that have not previously been examined for BHPDs, resulted in the identification of 63 different BHPDs falling into five different BHPD types: (i) nucleoside BHPDs, (ii) BHT, BHpentol, and BHhexol, and derivatives thereof, (iii) cyclitol ethers and precursors, (iv) carbamoyl BHPDs, and (v) aminotriols and ‐tetrols. Some of these BHPDs have not been previously identified in APB. The distribution of BHPDs varied to a large extent and did not follow the phylogenetic/taxonomical classification of APBs.

(4) These results were in good agreement with those obtained from Rohmer degradation on intact cells. These latter data allowed accurate assessment of the degree of methylation at C‐2 and C‐3 of ring A of the BHPDs, which showed a 1–2 orders of magnitude lower degree of methylation at C‐2 of BHPDs than for C_30_ hopanoids and tetrahymanol, with important implications for the interpretation of the molecular fossil record.

(5) Our results show that for the 54 studied APB species, the presence of BHPD biosynthetic genes, often organized in a biosynthetic gene cluster, in all cases results in actual production of BHPDs, indicating that the presence of BHPD genes is a good predictor for the actual production of BHPDs. However, the presence of genes encoding proteins that result in methylation at C‐2 and C‐3 of BHPDs does not always lead to the production of methylated BHPDs, complicating the interpretation of the presence of the *hpnP* and *hpnR* genes in their genomes.

(6) Overall, the results of this study raise caution for the interpretation of the geological record of hopanoids. BHPD concentrations in APB species that do produce hopanoids can vary by two orders of magnitude, which is not directly related to a specific phylogenetic group. The consequence of these findings is that in the environment the origin of the BHPDs may be biased towards specific species that produce relatively high amounts of BHPDs. Additionally, the BHPD biosynthetic gene cluster is not evenly distributed over taxonomic/phylogenetic groups as one would expect when this trait was only primarily obtained by vertical gene transfer and BHPD synthesis would be a conservative trait. This means that one cannot predict the production of specific BHPDs in one species by analyzing a closely related species. It will be even harder to predict based on the presence or absence of BHPDs in extant microbes if related species in the geological past possessed BHPDs. This complicates the use of BHPDs as biomarkers in environmental and palaeontological studies. Specifically, the rapid loss of *hpnP* during the evolution of certain genera of APB furthermore illustrates that molecular paleontologists should perhaps be more careful in applying the guiding principle “the present is the key to the past” on geological timescales relevant for OAEs (i.e., 100 Ma).

## Conflicts of Interest

The authors declare no conflicts of interest.

## Supporting information


**Figure S1:** MS^2^ spectrum of a novel BHPD, N‐phenylalanyl 35‐aminotriol (Vd), identified in *Variibacter gotjawalensis*.

**Figure S2:** Phylogenetic tree of the GyrB protein encoded by genomes of *Spingomona*s spp. in the NCBI databank of protein reference sequences. Species with genomes containing *shc* are marked in yellow, revealing the highly scattered occurrence of the ability of BHPD biosynthesis in this genus. The six *Spingomona*s spp. that were tested for actual BHPD production are underlined.

**Table S1:** MRM conditions used for the detection of hopenes, hopanols and tetrahymanol (as their TMS derivatives) in the Rohmer degradation products of APBs.

**Table S2:** Genes involved in the biosynthesis of BHPs in APB and the corresponding protein sequences used in PSI‐BLAST searches (see Tables S4 and S5).

**Table S3:** Accession numbers of the 16S rRNA gene sequences of APB and the outgroup used to construct the tree shown in Figures 2a and 3a.

**Table S4:** The information (i.e., accession lists of hits, parameter settings, and acquisition date) of all the PSI‐BLAST searches that were performed to generate the data listed in Table 2. Because of the large number of APB genomes (> 6500) the PSI‐BLAST searches of GyrB, Shc, HpnH, HpnP, and HpnR were in most cases splitted according to the four major orders within the APB.

**Table S5:** The information (i.e., accession lists of hits, parameter settings, and acquisition date) of all the PSI‐BLAST searches that were performed to assess the presence/absence of *hpn* genes in the 54 species of APB studies. These data form the basis for Figures 1b and 2b.

**Table S6:** Distribution of BHPDs (in % of total) detected in APB. Roman numerals refer to structures shown in Figure 5. These data form the basis for Figure 3.

## Data Availability

The data that support the findings of this study are openly available in the published and the Supporting Information provided.
