# Supplementary material for: A Comprehensive Study of Biohopanoid Production in Alphaproteobacteria: Biosynthetic, Chemotaxonomical, and Geobiological Implications
Source: Geobiology. 2025 Nov 4;23(6):e70038. doi: 10.1111/gbi.70038 (PMC12583986; doi:10.1111/gbi.70038)
Supplement: Supplementary file 1 — Figure S1: MS2 spectrum of a novel BHPD, N‐phenylalanyl 35‐aminotriol (Vd), identified in Variibacter gotjawalensis. [file GBI-23-e70038-s003.pdf]

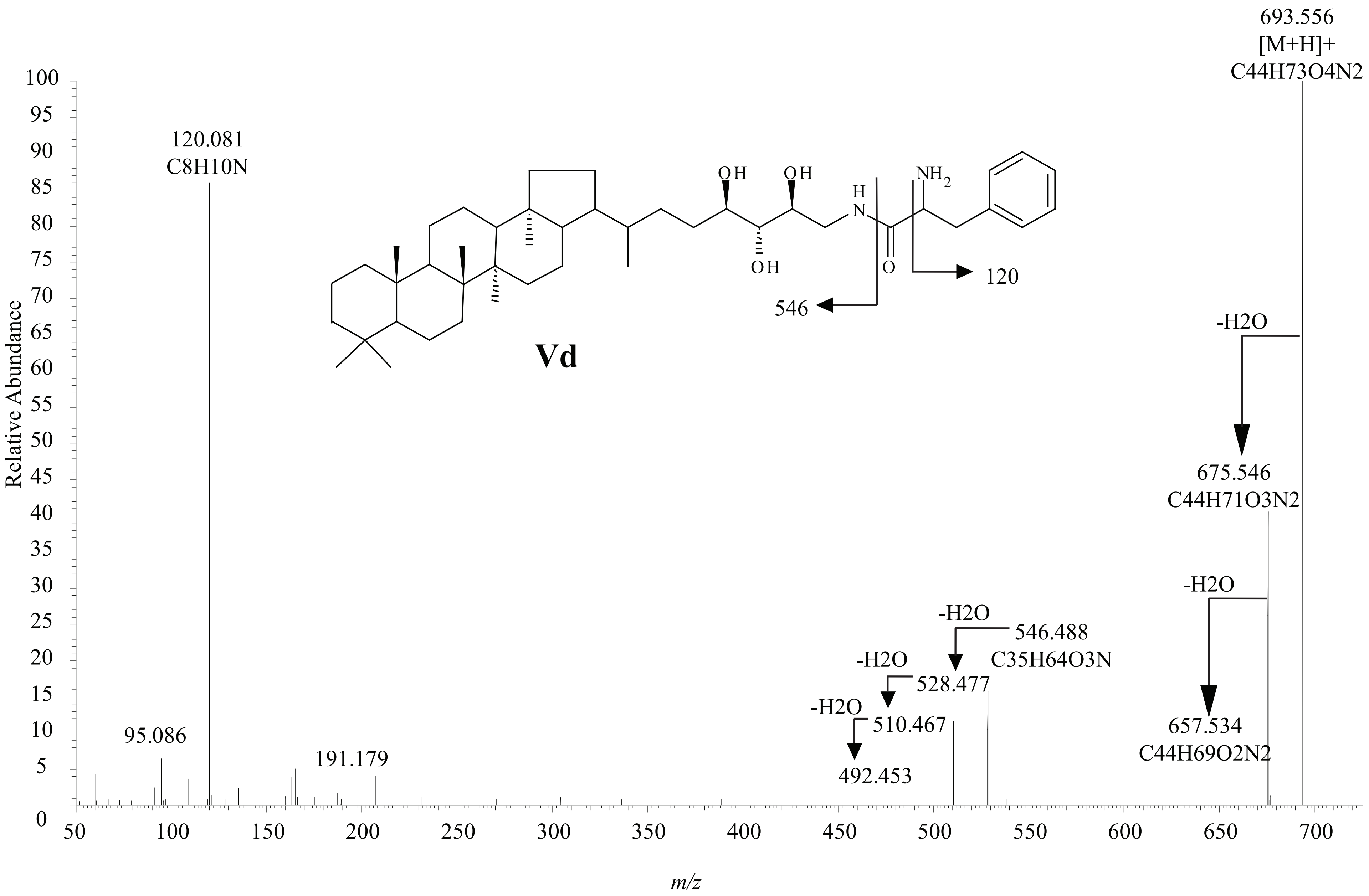


Figure S1: Mass spectrum of N-phenylalanyl 35-aminotriol (Vd) identified in *Variibacter gotjawalensis*.
