# Supplementary figures and images for: A Comprehensive Study of Biohopanoid Production in Alphaproteobacteria: Biosynthetic, Chemotaxonomical, and Geobiological Implications
Source: Geobiology. 2025 Nov 4;23(6):e70038. doi: 10.1111/gbi.70038 (PMC12583986; doi:10.1111/gbi.70038)

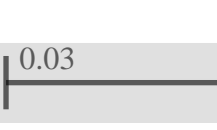

Supplement: Supplementary file 2 — Figure S2: Phylogenetic tree of the GyrB protein encoded by genomes of Spingomonas spp. in the NCBI databank of protein reference sequences. Species with genomes containing shc are marked in yellow, revealing the highly scattered occurrence of the ability of BHPD biosynthesis in this genus. The six Spingomonas spp. that were tested for actual BHPD production are underlined. [file GBI-23-e70038-s002.pdf]
