# Supplementary material for: A Comprehensive Study of Biohopanoid Production in Alphaproteobacteria: Biosynthetic, Chemotaxonomical, and Geobiological Implications
Source: Geobiology. 2025 Nov 4;23(6):e70038. doi: 10.1111/gbi.70038 (PMC12583986; doi:10.1111/gbi.70038)
Supplement: Supplementary file 3 — Table S1: MRM conditions used for the detection of hopenes, hopanols and tetrahymanol (as their TMS derivatives) in the Rohmer degradation products of APBs. [file GBI-23-e70038-s005.docx]

| **Table S1: MRM conditions used for the detection of hopenes, hopanols and tetrahymanol (as their TMS derivatives) in the Rohmer degradation products of APBs.** | | | | |
| --- | --- | --- | --- | --- |
| **Time window**  **(#)** | **Compound** | **Precursor ion (*m/z*)** | **Product ion** | **Retention**  **time (min)** |
| **1** | Hop-22(29)-ene | 410.4 | 191.2 | 35.7 |
| (2 transitions) | 2-Me-hop-22(29)-ene | 424.4 | 205.2 | 35.7 |
|  | 3-Me-hop-22(29)-ene | 424.4 | 205.2 | ca. 37 |
|  | Tetrahymanol | 410.4 | 191.2 | 37.3 |
|  | 2-Me-tetrahymanol | 424.4 | 205.2 | 37.4 |
|  | Diplopterol | 410.4 | 191.2 | 37.4 |
|  | 2-Me-diplopterol | 424.4 | 205.2 | 37.5 |
| **2** | Hopan-30-ol | 500.4 | 191.2 | 38.2 |
| (3 transitions) | 2-Me-hopan-30-ol | 514.5 | 205.2 | 38.2 |
|  | 3-Me-tetrahymanol | 424.4 | 205.2 | ca. 39 |
|  | 3-Me-diplopterol | 424.4 | 205.2 | ca. 39 |
| **3** | 3-Me-hopanol | 514.5 | 205.2 | 39.8 |
| (3 transitions) | Homohopan-31-ol | 514.5 | 191.2 | 39.9 |
|  | 2-Me-homohopan-31-ol | 528.5 | 205.2 | 39.9 |
| **4** | 22S bishomohopan-32-ol | 528.5 | 191.2 | 40.8 |
| (3 transitions) | 22S 2-Me-bishomohopan-32-ol | 542.5 | 205.2 | 40.8 |
|  | 3-Me-homohopan-31-ol | 528.5 | 205.2 | 41.5 |
|  | 22R bishomohopan-32-ol | 528.5 | 191.2 | 41.6 |
|  | 22R 2-Me-bishomohopan-32-ol | 542.5 | 205.2 | 41.6 |
|  | 3-Me-C32(22S)-hopanol | 542.5 | 205.2 | 42.4 |
|  | 3-Me-C32(22R)-hopanol | 542.5 | 205.2 | 43.2 |
