# Supplementary material for: A Comprehensive Study of Biohopanoid Production in Alphaproteobacteria: Biosynthetic, Chemotaxonomical, and Geobiological Implications
Source: Geobiology. 2025 Nov 4;23(6):e70038. doi: 10.1111/gbi.70038 (PMC12583986; doi:10.1111/gbi.70038)
Supplement: Supplementary file 4 — Table S2: Genes involved in the biosynthesis of BHPs in APB and the corresponding protein sequences used in PSI‐BLAST searches (see Tables S4 and S5). [file GBI-23-e70038-s006.docx]

| **Table S2: Genes involved in the biosynthesis of BHPs in APB and the corresponding protein sequences used in PSI Blast searches** | | | | | |
| --- | --- | --- | --- | --- | --- |
| **Gene** | **Annotation** | **Function** | **Reference** | **Protein accession No.**^a^ | **Locus tag**^a^ |
| *dxs* | 1-deoxy-D-xylulose-5-phosphate synthase | Catalyzes the condensation of pyruvate and glyceraldehyde 3-phosphate to yield 1-deoxy-D-xylulose-5-phosphate (DXP) | Zhao et al. (2013) | WP_011156487.1  WP_011240977.1 | RPAL_RS05130  ZMO_RS05150 |
| *dxr* | 1-deoxy-D-xylulose 5-phosphate reducto­isomerase | Catalyzes the NADP-dependent rearrangement and reduction of DXP to 2-C-methyl-D-erythritol 4-phosphate (MEP) | Zhao et al. (2013) | WP_012496356.1  WP_011240977.1 | RPAL_RS16105  ZMO_RS05150 |
| *ispD* | 2-C-methyl-D-erythritol 4-phosphate cytidylyltransferase | Catalyzes the formation of 4-diphosphocytidyl-2-C-methyl-D-erythritol from CTP and ME. | Zhao et al. (2013) | WP_012496039.1  WP_017466418.1 | RPAL_RS14170  ZMO_RS05040 |
| *ispE* | 4-diphosphocytidyl-2-C-methyl-D-erythritol kinase | Catalyzes the phosphorylation of the position 2 hydroxy group of 4-diphosphocytidyl-2-C-methyl-D-erythritol | Zhao et al. (2013) | WP_041810255.1  WP_011241005.1 | RPAL_RS06150  ZMO_RS05295 |
| *ispF* | 2-C-methyl-D-erythritol 2,4-cyclodiphosphate synthase | Catalyzes the conversion of 4-diphosphocytidyl-2-C-methyl-D-erythritol 2-phosphate (CDP-ME2P) to 2-C-methyl-D-erythritol 2,4-cyclodiphosphate (ME-CPP) | Zhao et al. (2013) | WP_012496039.1  WP_017466418.1 | RPAL_RS14170  ZMO_RS05040 |
| *ispG* | 4-hydroxy-3-methylbut-2-en-1-yl diphosphate synthase | Converts 2-C-methyl-D-erythritol 2,4-cyclodiphosphate (ME-2,4cPP) into 1-hydroxy-2-methyl-2-(E)-butenyl 4-diphosphate, using flavodoxin as the reducing agent | Zhao et al. (2013) | WP_012494214.1  WP_011240135.1 | RPAL_RS02635  ZMO_RS00770 |
| *ispH* | 4-hydroxy-3-methylbut-2-en-1-yl diphosphate reductase | Converts 1-hydroxy-2-methyl-2-(E)-butenyl 4-diphosphate into isopentenyl diphosphate (IPP) and dimethylallyl diphosphate (DMAPP) | Zhao et al. (2013) | WP_012497128.1  WP_011240741.1 | RPAL_RS21055  ZMO_RS03915 |
| *ispA* | Farnesyl diphosphate synthase | Production of farnesyl diphosphate from IPP and DMAPP | Thulasiram and Poulter (2006) | WP_012494216.1  WP_011240722.1 | RPAL_RS02660  ZMO_RS03820 |
| *hpnA* | Hopanoid-associated sugar epimerase | Not established, possibly involved in side-chain formation | Perzl et al. (1998);  Schmerk et al. (2015) | WP_011240733.1  WP_012253485.1  WP_006483842.1 | ZMO_RS03875  MEXAM1_RS08685  K562_RS31040 |
| *hpnB* | Hopanoid-associated glycosyl transferase | Not established, possibly involved in side-chain formation | Perzl et al. (1998);  Schmerk et al. (2015) | WP_011240734.1  WP_012493627.1 | ZMO_RS03880  K562_RS31050 |
| *hpnC* | Squalene/phytoene synthase | Conversion of presqualene diphosphate into hydroxysqualene | Pan et al. (2015) | WP_011159281.1  WP_011240735.1  WP_038965749.1  WP_003604029.1 | RPAL_RS21100  ZMO_RS03885  AAV28_RS11515  MEXAM1_RS08690 |
| *hpnD* | Squalene/phytoene synthase | Production of presqualene diphosphate from farnesyl diphosphate | Pan et al. (2015) | WP_012497135.1  WP_011240736.1  WP_011085786.1  WP_003604030.1  WP_006485179.1 | RPAL_RS21095  ZMO_RS03890  AAV28_RS11520  MEXAM1_RS08695  K562_RS31530 |
| *hpnE* | Amine oxidoreductase | Reduction of hydroxysqualene | Pan et al. (2015) | WP_012497134.1  WP_011240737.1  WP_011085787.1  WP_012752633.1  WP_012493654.1 | RPAL_RS21090  ZMO_RS03895  AAV28_RS11525  MEXAM1_RS08700  K562_RS31525 |
| *shc (hpnF)* | Squalene hopene cyclase | Formation of diploptene from cyclization of squalene | Reipen et al. (1995) | WP_012497133.1  WP_011240738.1  WP_011085788.1  WP_003604034.1  WP_006482092.1 | RPAL_RS21085  ZMO_RS03900  AAV28_RS11530  MEXAM1_RS08705  K562_RS31520 |
| *hpnH* | Hopanoid-associated radical SAM superfamily protein | Addition of adenosine to hopane skeleton | Bradley et al. (2010); Welander et al. (2012);  Schmerk et al. (2015);  Sato et al. (2020) | WP_011159273.1  WP_011240739.1  WP_011085790.1  WP_003601722.1  WP_012493627.1 | RPAL_RS21060  ZMO_RS03905  AAV28_RS11540  MEXAM1_RS17455  K562_RS31060 |
| *hpnG* | Hopanoid-associated nucleosidase | Removal of adenine from adenosylhopane | Bradley et al. (2010); Welander et al. (2012);  Schmerk et al. (2015) | WP_012497132.1  WP_011240740.1  WP_028172326.1  WP_012753333.1  WP_006494145.1 | RPAL_RS21080  ZMO_RS03910  AAV28_RS11535  MEXAM1_RS17460  K562_RS31515 |
| *hpnI* | Hopanoid-associated glycosyl transferase | Transfer of acetylglucosamine from UDP-acetylglucosamine to BHT | Schmerk et al. (2015) | WP_011240825.1  WP_003601711.1  WP_020979909.1 | ZMO_RS04350  MEXAM1_RS17430  K562_RS13435 |
| *hpnJ* | Hopanoid-associated radical SAM superfamily protein | Ring contraction to generate BHT cyclitol ether | Schmerk et al. (2015) | WP_026059405.1  WP_003601712.1  WP_369718636.1 | ZMO_RS04355  MEXAM1_RS17435  K562_RS13430 |
| *hpnK* | Hopanoid biosynthesis associated protein | Deacetylation of BHT acetylglucosamine | Schmerk et al. (2015) | WP_011240827.1  WP_003601714.1  WP_006493052.1 | ZMO_RS04360  MEXAM1_RS17440  K562_RS13425 |
| *hpnL* |  | Not established | Schmerk et al. (2015) | WP_011240828.1  WP_003601707.1  K562_RS13425 | ZMO_RS04365  MEXAM1_RS17420  K562_RS13420 |
| *hpnM* | ABC transporter membrane protein | Possibly involved in transport of hopanoids | Schmerk et al. (2015); Rubiano-Labrador et al. (2015) | WP_011240742.1  WP_006485934.1 | ZMO_RS03920  K562_RS31500 |
| *hpnN* | hopanoid-associated RND transporter | Transport of hopanoids to the outer membrane | Doughty et al. (2011) | WP_012497127.1  WP_011241353.1  WP_011085792.1  WP_003601720.1  WP_012493652.1 | RPAL_RS21050  ZMO_RS07175  AAV28_RS11550  MEXAM1_RS17450  K562_RS31505 |
| *hpnO* | aminotransferase | Replaces the OH group at C-35 with an NH_2_ group. | Welander et al. (2012); Liu et al. (2014) | WP_011159270.1  WP_011085794.1 | RPAL_RS21045  AAV28_RS11560 |
| *hpnP* | Hopanoid C-2 methylase HpnR | Introduces a methyl group at position C-2 in a BHP or C_30_ hopanoid | Welander et al. (2010) | WP_012497138.1  AAV28_RS11515  WP_003597531.1 | RPAL_RS21125  AAV28_RS11485  MEXAM1_RS20755 |
| *hpnR* | Hopanoid C-3 methylase HpnR | Introduces a methyl group at position C-3 in a BHP or C_30_ hopanoid | Welander and Summons (2012) | WP_010960068.1  WP_014457164.1 | MCA_RS03630  FCN51_RS11225 |
| *hpnX* | Sterol desaturase | Possibly responsible for the introduction of a double bond | Tushar et al. (2014) | WP_037236121.1 | T281_RS06660 |

^a^ Colour codes refer to various model proteobacteria: Red = *Rhodopseudomonas palustris* TIE-1 (assembly No. GCF_000020445.1); Green = *Zymomonas mobilis* ZM4 (GCF_004168305.2); Yellow = *Bradyrhizobium diazoefficiens* USDA 110 (GCF_001642675.1); Grey = *Methylorubrum extorquens* AM1 (GCF_000022685.1); Blue = *Burkholderia cenocepacia* K56-2 (GCF_014357995.1); Gold = *Methylococcus capsulatus* Bath (GCF_000008325.1); Purple = *Acetobacter pasteurianus* DSM 3509 (GCF_003850805.1); Pink = *Rhodomicrobium udaipurense* JA643 (GCF_000636015.1
