# Supplementary material for: A Comprehensive Study of Biohopanoid Production in Alphaproteobacteria: Biosynthetic, Chemotaxonomical, and Geobiological Implications
Source: Geobiology. 2025 Nov 4;23(6):e70038. doi: 10.1111/gbi.70038 (PMC12583986; doi:10.1111/gbi.70038)
Supplement: Supplementary file 5 — Table S3: Accession numbers of the 16S rRNA gene sequences of APB and the outgroup used to construct the tree shown in Figures 2a and 3a. [file GBI-23-e70038-s007.docx]

| **Table S3: Accession numbers of the 16S rRNA gene sequences of APB and the outgroup used to construct the tree shown in Fig. 2a and 3a.** | | | |
| --- | --- | --- | --- |
| **Order** | **Species** | **DSM culture number** | **Accession number** |
| *Hyphomicrobiales* | *Afipia broomeae B91-007286* | 7327^T^ | NR_029200.1 |
|  | *Beijerinckia indica* *B.102.C* | 591 | AB119196.1 |
|  | *Bradyrhizobium elkanii USDA61* | 11554^T^ | U35000.3 |
|  | *Chelatococcus reniformis B2974* | 105737^T^ | NR_152704.1 |
|  | *Ensifer sojae* | 26426^T^ | NR_117540.1 |
|  | *Enterovirga rhinocerotis* | 25903^T^ | NR_149206.1 |
|  | *Hyphomicrobium facile* | 1565^T^ | NR_036978.1 |
|  | *Methylobacterium aquaticum* | 16371^T^ | NR_025631.1 |
|  | *Methylobacterium oxalidis* | 24028^T^ | NR_113302.1 |
|  | *Methylobacterium soli* | 21955^T^ | NR_116473.1 |
|  | *Methylocella palustris* | n.a. | NR_027561.1 |
|  | *Methyloferula stellata AR4* | 22108^T^ | NR_117077.1 |
|  | *Methylorubrum rhodesianum D2_2* | 103741 | NR_041028.1 |
|  | *Microvirga massiliensis JC119* | 26813^T^ | NR_144703.1 |
|  | *Rhizobium tropici CIAT 899* | 11418^T^ | NR_026067.1 |
|  | *Rhodoligotrophos appendicifer 120-1* | 23582^T^ | NR_113322.1 |
|  | *Rhodomicrobium vannielii ATH 3.1.1* | 162^T^ | NR_117027.1 |
|  | *Rhodoplanes elegans* | 11907^T^ | D25311.2 |
|  | *Rhodopseudomonas parapalustris* | 130 | NR_122098.1 |
|  | *Roseiarcus fermentans Pf56* | 24875^T^ | NR_134158.1 |
|  | *Roseibium album CECT5095* | 18320^T^ | NR_042378.1 |
|  | *Roseibium marinum mano 18* | 17023^T^ | NR_043040.1 |
|  | *Variibacter gotjawalensis* | 29671^T^ | NR_134225.1 |
| *Rhodospirillales* | *Acetobacter pasteurianus* | 3509^T^ | NR_026107.1 |
|  | *Acidocella aminolytica* | 11237^T^ | NR_025849.1 |
|  | *Asaia siamensis S60-1* | 15972^T^ | NR_024738.1 |
|  | *Azospirillum brasilense SP 7* | 1690^T^ | NR_042845.1 |
|  | *Enhydrobacter aerosaccus* | 8914^T^ | AB641398.1 |
|  | *Gluconacetobacter diazotrophicus PA5* | 5601^T^ | NR_027591.1 |
|  | *Gluconobacter oxydans CN 1221* | 2003 | NR_026118.1 |
|  | *Hypericibacter terrae R5913* | 109816^T^ | MG271952.1 |
|  | *Komagataeibacter europaeus JK2* | 13110 | NR_026513.1 |
|  | *Komagataeibacter xylinus R-2277* | n.a. | NR_036787.1 |
|  | *Kozakia baliensis* | 14400^T^ | NR_024773.1 |
|  | *Limimonas halophila IA16* | 25584^T^ | NR_109490.1 |
|  | *Magnetospirillum fulvum 1360* | 113T | NR_025836.1 |
|  | *Nitrospirillum amazonense Y-1* | 2787^T^ | NR_119099.1 |
|  | *Pelagibius litoralis* | 21314^T^ | NR_043785.1 |
|  | *Rhodopila globiformis* | 161^T^ | MW326760.1 |
|  | *Rhodospirillum rubrum S 1* | 467^T^ | NR_074249.1 |
|  | *Roseomonas pecuniae N75* | 25622^T^ | NR_104544.1 |
|  | *Skermanella aerolata 5416T-32* | 18479^T^ | NR_043929.1 |
|  | *Tistlia consotensis USBA 355* | 21585^T^ | NR_116437.1 |
| *Sphingomonadales* | *Novosphingobium acidiphilum FSW 06-204d* | 19966 | NR_116278.1 |
|  | *Novosphingobium nitrogenifigens Y 88* | 19370^T^ | NR_043857.1 |
|  | *Novosphingobium rosa* | 7285^T^ | NR_104206.1 |
|  | *Sphingobium estronivorans AXB* | 102173^T^ | KM925003.2 |
|  | *Sphingomonas alpina S8-3* | 22537^T^ | NR_117230.1 |
|  | *Sphingomonas changbaiensis V2M44* | 25652^T^ | NR_116415.1 |
|  | *Sphingomonas formosensis CC-Nfb-2* | 24164^T^ | NR_117828.1 |
|  | *Sphingomonas haloaromaticamans A175* | 13477^T^ | NR_044902.1 |
|  | *Sphingomonas mali Y-347* | 10565^T^ | NR_113762.1 |
|  | *Stakelama sediminis* | 27203^T^ | NR_116171.1 |
| *Micropepsales* | *Rhizomicrobium palustre A48* | 19867^T^ | NR_112186.1 |
| **Outgroup** |  |  |  |
| *Methylococcales* | *Methylococcus capsulata* |  | JN166982.1 |
